# Design and Cross-Cultural Invariance of the COVID-19 Vaccine Conspiracy Beliefs Scale (COVID-VCBS) in 13 Latin American Countries

**DOI:** 10.3389/fpubh.2022.908720

**Published:** 2022-06-14

**Authors:** Tomás Caycho-Rodríguez, Pablo D. Valencia, José Ventura-León, Lindsey W. Vilca, Carlos Carbajal-León, Mario Reyes-Bossio, Michael White, Claudio Rojas-Jara, Roberto Polanco-Carrasco, Miguel Gallegos, Mauricio Cervigni, Pablo Martino, Diego Alejandro Palacios, Rodrigo Moreta-Herrera, Antonio Samaniego-Pinho, Marlon Elías Lobos-Rivera, Andrés Buschiazzo Figares, Diana Ximena Puerta-Cortés, Ibraín Enrique Corrales-Reyes, Raymundo Calderón, Bismarck Pinto Tapia, Walter L. Arias Gallegos, Olimpia Petzold

**Affiliations:** ^1^Facultad de Ciencias de la Salud, Universidad Privada del Norte, Lima, Peru; ^2^Facultad de Estudios Superiores Iztacala, Universidad Nacional Autónoma de Mexico, Tlanepantla de Baz, Mexico; ^3^South American Center for Education and Research in Public Health, Universidad Norbert Wiener, Lima, Peru; ^4^Facultad de Psicología, Universidad Peruana de Ciencias Aplicadas, Lima, Peru; ^5^Facultad de Ciencias Humanas y Educación, Universidad Peruana Unión, Lima, Peru; ^6^Departamento de Psicología, Facultad de Ciencias de la Salud, Universidad Católica del Maule, Talca, Chile; ^7^Cuadernos de Neuropsicología, Rancagua, Chile; ^8^Programa de Pós-Graduação em Psicología, Pontificia Universidade Católica de Minas Gerais, Belo Horizonte, Brazil; ^9^Consejo Nacional de Investigaciones Científicas y Técnicas, Buenos Aires, Argentina; ^10^Facultad de Psicología, Universidad Nacional de Rosario, Rosario, Argentina; ^11^Centro Interdisciplinario de Investigaciones en Ciencias de la Salud y del Comportamiento, Universidad Adventista del Plata, Consejo Nacional de Investigaciones Científicas y Técnicas, Rosario, Argentina; ^12^Centro de Desarrollo Humano, Universidad Mariano Gálvez, Guatemala, Guatemala; ^13^Escuela de Psicología, Pontificia Universidad Católica del Ecuador, Ambato, Ecuador; ^14^Carrera de Psicología, Facultad de Filosofía, Universidad Nacional de Asunción, Asunción, Paraguay; ^15^Facultad de Ciencias Sociales, Escuela de Psicología, Universidad Tecnológica de El Salvador, San Salvador, El Salvador; ^16^Centro de Estudios Adlerianos, Instituto Alfred Adler Uruguay, Montevideo, Uruguay; ^17^Programa de Psicología, Universidad de Ibagué, Ibagué, Colombia; ^18^Servicio de Cirugía Maxilofacial, Hospital General Universitario Carlos Manuel de Céspedes, Universidad de Ciencias Médicas de Granma, Bayamo, Cuba; ^19^Carrera de Psicología, Facultad de Ciencias de la Salud, Universidad del Valle de Mexico, Ciudad de Mexico, Mexico; ^20^Carrera de Psicología, Universidad Católica Boliviana San Pablo, La Paz, Bolivia; ^21^Departamento de Psicología, Universidad Católica San Pablo, Arequipa, Peru; ^22^Lone Star College-Conroe Center, Conroe, TX, United States; ^23^Psychosomatic and Psycho-Oncological Research Unit, Université Libre de Bruxelles, Brussels, Belgium

**Keywords:** conspiracy beliefs, COVID-19, invariance, Latin America, vaccines

## Abstract

**Aims:**

Over the past 2 years, the vaccine conspiracy beliefs construct has been used in a number of different studies. These publications have assessed the determinants and outcomes of vaccine conspiracy beliefs using, in some cases, pooled data from different countries, and compared the results across these contexts. However, studies often do not consider measurement invariance as a necessary requirement for comparative analyses. Therefore, the aim of this study was to develop and evaluate the cross-cultural MI of the COVID-19 Vaccine Conspiracy Beliefs Scale (COVID-VCBS) in 12 Latin American countries.

**Methods:**

Confirmatory factor analysis, item response theory analysis and alignment method were applied to test measurement invariance in a large number of groups.

**Results:**

The COVID-VCBS showed robust psychometric properties and measurement invariance for both factor loadings and crosstabs. Also, a higher level of acceptance of conspiracy beliefs about vaccines is necessary to respond to higher response categories. Similarly, greater acceptance of conspiracy beliefs about COVID-19 vaccines was related to a lower intention to be vaccinated.

**Conclusion:**

The results allow for improved understanding of conspiracy beliefs about COVID-19 vaccines in the countries assessed; furthermore, they provide researchers and practitioners with an invariant measure that they can use in cross-cultural studies in Latin America. However, further studies are needed to test invariance in other countries, with the goal of developing a truly international measure of conspiracy beliefs about COVID-19 vaccines.

## Introduction

The current COVID-19 pandemic has had unprecedented negative health, social, and economic impacts (1) and has led to, as of January 10, 2022, more than 300 million confirmed cases of COVID-19, with more than 5 million deaths resulting from the disease (2). In this context, timely development of effective vaccines and their equitable distribution is the best strategy to reduce the negative impact of COVID-19 (3). However, for this to reach its full effect, vaccination programs need to achieve herd immunity. Evidence from previous pandemics showed that vaccinating 80–90% of the population established herd immunity (4). For COVID-19 variants of concern such as Alpha, the threshold for herd immunity is around 80% (5), whereas it may be higher for variants such as Delta (6). These estimates may vary by country and infection rate (7). However, a major barrier to achieving this goal is the high prevalence vaccine rejection, which has been a problem that has appeared in previous pandemics, such as the H1N1 outbreak (8) and which hinders year after year the prevention of seasonal influenza (9). It has recently been indicated that refusal or hesitancy to receive a COVID-19 vaccination showed a level of <30% of the general population, where the lowest refusal rates were found in Ecuador (3%), Malaysia (5.7%), Indonesia (6.7%) and China (8.7%); whereas, the highest rates of refusal or hesitancy were found in Kuwait (76.4%), Jordan (71.6%), Italy (46.3), Russia (45.1%), Poland (43.7%), the United States (43.1%) and France (41.4%) (10). Refusal or hesitancy to accept the vaccine is more common in developing countries (11), such as those in Latin America. Thus, a study in six Latin American countries (Argentina, Brazil, Chile, Colombia, Mexico, and Peru) reported that only 59% of respondents would accept a vaccination against COVID-19 (12); while other research conducted in Latin America and the Caribbean indicated that about 81.2% of participants were afraid of adverse effects from the COVID-19 vaccine (13).

Increased refusal to receive a vaccine is related to misinformation about the importance, safety or efficacy of vaccines (14). The fear and uncertainty that have accompanied the COVID-19 pandemic have fostered a rapid spread of misinformation, along with the adoption of conspiracy beliefs in a significant portion of the world's population (15–17). Conspiracy beliefs can be defined as a set of false beliefs in which an event is believed to be an organized plot by multiple actors working together with a defined goal, which is often illegal and secret (18).

Specifically, doubts or refusals to be vaccinated against COVID-19 have been related to the belief that vaccine production was too fast, that the risks of vaccination outweigh the benefits, and that vaccines are a strategy of large pharmaceutical companies to increase their profits (19). In addition, cover-ups of the health risks associated with vaccination and that vaccination is part of a plot to implant microchips or quantum dot software to control and track the world's population have been mentioned (17, 20). Other erroneous claims suggest that the SARS-CoV-2 virus was created in a lab as a biological weapon and that messenger RNA vaccines cause infertility (15, 21).

The presence of conspiracy beliefs is negatively associated with adherence to preventive behaviors against COVID-19 (22, 23). It has been suggested that people with conspiratorial beliefs about COVID-19 tend to feel alienated and distrustful; in addition, they are more focused on their own personal well-being, and less concerned about the well-being of others (24). The distrust felt by people who support conspiracy beliefs toward vaccines would be related to negative attitudes toward powerful groups, such as medical or political institutions (25). In a sense, conspiracy beliefs seem to protect people who support these ideas from the anxiety and distress associated with death, leading them to deny the problem of COVID-19 infection as a coping mechanism and, therefore, to refuse the vaccine (26).

Despite concerns about the increase in fake news about vaccines and recent outbreaks of new variants of COVID-19, there is a paucity of research examining conspiracies about COVID-19 vaccines in Latin America (27). In this sense, having a standardized and validated measure for the Latin American context will allow for a better understanding of the impact of vaccine conspiracy beliefs on rates of refusal, hesitancy, and acceptance of COVID-19 vaccines. There are measures that assess conspiracy beliefs generally, not necessarily related to vaccines (28–30) and others associated with vaccines (31, 32). However, as far as is known from the literature, there is no scale that explicitly assesses conspiracy beliefs about COVID-19 vaccines invariantly (i.e., in the same way) across a diverse group of Latin American countries.

Most studies that design and evaluate the psychometric properties of measurement instruments are based on Classical Test Theory (CTT) criteria (33). This has also been observed in the psychometric evaluation of other scales assessing conspiratorial beliefs in general and about vaccines (29, 30, 32). Although CTT is an established approach for the construction and psychometric evaluation of measurement instruments in psychology and other sciences, it also has some methodological limitations. Thus, for example, scale and item statistics, such as item difficulty and reliability, depend on the sample; whereas, the score an individual obtains is influenced by the characteristics of the test (34). Thus, in addition to CTT, Item Response Theory (IRT) is often used for the development and evaluation of measurement instruments (35). IRT models allow for describing the relationship between an individual's response to an item, the individual's ability, and item characteristics such as difficulty and discrimination; moreover, IRT models are useful for dichotomous and polytomous variables (36, 37). One of the main advantages of IRT models is that the model parameters are independent of the study sample; in addition, standard errors can be calculated separately according to each person's ability (34).

The application of IRT models may vary according to the item scoring methods (dichotomous and polytomous) or the number of factors assessed by the measure (unidimensional and multidimensional). One of the most commonly used models for polytomous items is the Graduated Response Model (GRM) (38), which assumes that the response categories of an item can be ordered and uses the totality of information from each item response to better measure people (39). Despite their limitations, CTT procedures have the advantage that they can be performed without requiring very large samples; whereas IRT-based procedures require larger samples (34). Despite the advantages of IRT over CTT, there are also similarities. Thus, the difficulty and discriminative power indices of CTT, and the difficulty (b) and discrimination (a) parameters of ITR have the same meanings reciprocally. On the other hand, it has been suggested that IRT is derived from CTT, and that the latter is a very simple form of IRT. The widespread use of IRT models in Latin America is relatively recent and still limited in scope (40). However, the relationships between IRT and CTT are very useful for a better understanding of both approaches and to facilitate future well-informed applications (41).

While measures of conspiracy beliefs have been used in cross-cultural studies (42, 43) few have assessed cross-cultural measurement invariance (29). To our knowledge, previous studies that have assessed conspiracy beliefs about vaccines or antivaccine attitudes in different countries have not assessed cross-cultural measurement invariance (44). However, instruments that measure specific conspiracy beliefs, as in the case of vaccination, are highly associated with particular temporal and geographic contexts (29). In this sense, the comparability of an instrument measuring vaccine conspiracy beliefs across countries is a crucial problem and a growing need (45), as multinational studies can use the instrument to compare such beliefs across countries. Thus, measurement invariance (MI) is a necessary prerequisite for comparisons (46), since the absence of MI renders it impossible to know whether the presence or absence of differences in a construct (in this case, conspiracy beliefs about COVID-19 vaccines) between groups is a result of actual differences in the construct itself or is generated by the psychometric characteristics of the instrument. Having different meanings or interpretations of the construct, differences in the degree of social desirability, different responses to extreme items, having items that are more applicable to one group than to another, or an incorrect translation of the items can lead to an absence of MI (47). Although MI has been a suggested procedure as part of cross-cultural studies for years (48), instruments showing this evidence are still scarce (49).

Multigroup confirmatory factor analysis (MGCFA) has been the traditional method for assessing MI (50). MGCFA is based on the idea that comparisons between groups are appropriate as long as the instrument used measures exactly the same thing (51). For this, the MGCFA evaluates three levels of MI: (1) configural invariance, which evaluates whether or not the construct presents the same factor structure across groups; (2) metric invariance, where factor loadings, defined as the strength of the association between each item and the construct that it measures, are equal across groups and; (3) scalar invariance, which requires equality in factor loadings and item intercepts (which is understood as the expected score in people who present an average level in the measured construct) (52). The importance of the invariance analysis lies in determining whether the instrument measures the same construct in all groups. Likewise, the different levels of invariance indicate whether or not certain comparisons can be made. The presence of metric invariance would allow for the comparison of covariances and regression coefficients between groups; whereas, scalar invariance would also allow for the comparison of latent means (53). However, MGCFA has also received criticism (54, 55). Thus, it is considered that the MGCFA is too strict and rejects models that can be comparable, especially in cross-national studies comparing a large number of groups (51). The likelihood of not meeting some equivalence principles, and thus failing to achieve full MI, increases as the number of groups increases (50). Looking for an alternative to the MGCFA problem in cross-national studies, the Multi-Group Factor Analysis Alignment method (CFA-MIAL) was recently developed (54). The CFA-MIAL can estimate means without the need to restrict factor loadings and intercepts to equality between groups. This method evaluates the invariance of factor loadings and intercepts simultaneously and considers that they do not necessarily have to be identical between culturally diverse groups; moreover, it assumes that the number of non-invariant parameters and the degree of non-invariance can be minimal (54). This allows the CFA-MIAL to identify an invariant pattern among different groups and estimate means and variances considering the actual differences in factor loadings and intercepts. Although CFA-MIAL is a recent and little used procedure (54), it is an alternative that can automate and simplify MI (56).

Having an invariant measure across different countries is even more important considering that some countries may be more attentive to conspiracy beliefs than others (57). Thus, it is also suggested that conspiratorial thinking is not something strange, but rather it is frequent and, therefore, it is considered a cultural phenomenon shared by several countries. In this sense, conspiracy theories are found all over the world and, so far, no culture has been identified where conspiratorial beliefs do not exist (58). However, not all individuals or cultures accept conspiracy theories to the same extent. There are cultural differences in the acceptance to conspiracy theories, especially contexts of violence, exploitation or conflict between groups, such as civil unrest, high and low trust cultural groups, as well as power inequality between the elites and the common people (58). These characteristics are common in Latin American countries, where health systems have limited resources to cope with the pandemic, there is a higher prevalence of chronic diseases, late responses from populist governments, and high rates of poverty and inequality (59, 60). In addition, Latin America is the region with the worst satisfaction with democracy and with a high level of social protest, originated by inequality of opportunities and weak institutions (61).

While conspiracy beliefs about COVID-19 vaccines have been previously studied in the Latin American context, these have been assessed with measures of conspiracy beliefs about vaccines in general, but not specifically referring to COVID-19 (62). Therefore, the aim of this study was to develop and evaluate the cross-cultural MI of the COVID-19 Vaccine Conspiracy Beliefs Scale (COVID-VCBS) in 12 Latin American countries. Specifically, evidence of construct validity, internal consistency reliability, item discriminability and item difficulty, cross-cultural measurement invariance and of validity relative to other variables are evaluated. The evaluation of the evidence of construct validity was performed based on CTT procedures (factor structure, reliability, criterion validity) and IRT (evaluation of item parameters [slopes and thresholds] in the observed response patterns and the item characteristics curve). Evidence of validity with other variables was assessed by the association between conspiracy beliefs about COVID-19 vaccines and intention to be vaccinated against COVID-19. Previous studies have shown that rejection of conspiracy theories about COVID-19 in general and about COVID vaccines in particular are significant predictors of intention to be vaccinated against COVID-19 (25, 63–65). This is to be expected, as conspiracy beliefs about vaccines can increase vaccine hesitancy (31).

The present study contributes in several ways. First, it is the first and largest study to assess the cross-cultural measurement invariance of a measure of conspiracy beliefs about the COVID-19 vaccine in Latin America to date. Second, it assesses how these beliefs are related to fear of COVID-19. Third, it assesses and compares, for the first time in Latin America, conspiracy beliefs about vaccines in a group of 13 countries, offering additional information beyond that reported in a single country. Taken together, these findings mark important step toward developing a broader measure of conspiracy thinking about COVID-19 vaccines in Latin America.

## Method

### Design

The present study has an instrumental design (66) since it is focused on the validation and psychometric analysis of a measurement instrument.

### Participants

A total of 5,786 people residing in 13 Latin American countries (Argentina, Bolivia, Chile, Colombia, Cuba, Ecuador, El Salvador, Guatemala, Mexico, Paraguay, Peru, Uruguay, and Venezuela), selected by non-probability convenience sampling, participated. This type of sampling has been common among studies conducted during the COVID-19 pandemic (67, 68), due to restrictions on movement and interaction among individuals. To participate in the study, one had to be of legal age and provide informed consent.

The number of participants in each country ranged from 322 (Peru) to 747 (El Salvador). The sample size in each country for the present study is in accordance with the recommendations for confirmatory factor analysis and IRT, which were 300 and 375, respectively (67, 68). A total of 4,093 women and 1,687 men participated, with a mean age of 33.50 years old (SD = 13.4), where participants from Mexico were the youngest (M = 25, SD = 8.68); whereas, participants from Guatemala had the highest mean age (M = 44.04, SD = 13.62). In addition, most participants were single (61.23%), had a stable job (47.91%), and had completed university studies (47.08%). Finally, 52.56% reported not having had COVID-19; however, 71.62% and 86.54% indicated having had family and friends infected by COVID-19, respectively. Table 1 shows, in more detail, the sociodemographic information for each country.

**Table 1:** Sociodemographic Information of the Study Sample.

**Variable**	**Argentina (*n*= 369)**	**Bolivia (*n* = 567)**	**Chile** **(*n* = 453)**	**Colombia (*n* = 462)**	**Cuba** **(*n* = 334)**	**Ecuador** **(*n* = 438)**	**El Salvador (*n* = 747)**	**Guatemala (*n* = 420)**	**Mexico (*n* = 484)**	**Paraguay (*n* = 417)**	**Peru** **(*n* = 322)**	**Uruguay** **(*n* = 393)**	**Venezuela (*n* = 386)**
Gender	*n* (%)	*n* (%)	*n* (%)	*n* (%)	*n* (%)	*n* (%)	*n* (%)	*n* (%)	*n* (%)	*n* (%)	*n* (%)	*n* (%)	*n* (%)
Male	108 (29.8)	143 (25.2)	139 (30.7)	139 (30.1)	103 (30.8)	127 (29.0)	200 (26.8)	123 (29.3)	153 (31.6)	125 (30)	98 (30.4)	120 (30.5)	109 (28.2)
Female	255 (70.3)	421 (74.3)	314 (69.3)	322 (69.7)	231 (69.2)	311 (71.0)	546 (73.1)	297 (70.7)	331 (68.4)	292 (70)	224 (69.6)	273 (69.5)	276 (71.5)
Other	0 (0.0)	3 (0.5)	0 (0.0)	1 (0.2)	0 (0.0)	0 (0.0)	1 (0.1)	0 (0.0)	0 (0.0)	0 (0.0)	0 (0.0)	0 (0.0)	1 (0.3)
Marital status													
Single	198 (54.6)	247 (43.6)	264 (58.3)	368 (79.7)	194 (58.1)	289 (66)	502 (67.2)	172 (41.0)	407 (84.1)	258 (61.9)	251 (78)	227 (57.8)	166 (43.0)
Married	74 (20.4)	223 (39.3)	99 (21.9)	61 (13.2)	64 (19.2)	98 (22.4)	167 (22.4)	179 (42.6)	57 (11.8)	94 (22.5)	45 (14.0)	73 (18.6)	146 (37.8)
Cohabiting	45 (12.4)	31 (5.5)	61 (13.5)	23 (5.0)	65 (19.5)	22 (5.0)	40 (5.4)	31 (7.4)	7 (1.5)	45 (10.8)	21 (6.5)	63 (16.0)	22 (5.7)
Divorced	28 (7.7)	58 (10.2)	24 (5.3)	8 (1.7)	8 (2.4)	25 (5.7)	25 (3.4)	29 (6.9)	13 (2.7)	13 (3.1)	4 (1.2)	26 (6.6)	44 (11.4)
Widowed	18 (5.0)	8 (1.4)	5 (1.1)	2 (0.4)	3 (0.9)	4 (0.9)	13 (1.7)	9 (2.1)	0 (0.0)	7 (1.7)	1 (0.3)	4 (1.0)	8 (2.1)
Employment													
Stable job	165 (45.5)	239 (42.2)	253 (55.8)	124 (26.8)	190 (56.9)	154 (35.2)	367 (49.1)	258 (61.4)	158 (32.6)	254 (60.9)	119 (37.0)	264 (67.2)	227 (58.8)
Temporary job	59 (16.3)	159 (28.0)	64 (14.1)	104 (22.5)	28 (8.4)	103 (23.5)	122 (16.3)	87 (20.7)	88 (18.2)	85 (20.4)	122 (37.9)	51 (13.0)	58 (15.0)
Unemployed	87 (24.0)	150 (26.5)	125 (27.6)	218 (47.2)	114 (34.1)	159 (36.3)	218 (29.2)	44 (10.5)	226 (46.7)	70 (16.8)	76 (23.6)	68 (17.3)	67 (17.4)
Retired	52 (14.3)	19 (3.4)	11 (2.4)	16 (3.5)	2 (0.6)	22 (5.0)	40 (5.4)	31 (7.4)	12 (2.5)	8 (1.9)	5 (1.6)	10 (2.5)	34 (8.8)
Education													
University (complete)	159 (43.8)	418 (73.7)	266 (58.7)	120 (26.0)	167 (50.0)	189 (43.2)	170 (22.8)	267 (63.6)	128 (26.4)	269 (64.5)	135 (41.9)	176 (44.8)	260 (67.4)
University (incomplete)	137 (37.7)	85 (15)	106 (23.4)	127 (27.5)	152 (45.5)	140 (32.0)	262 (35.1)	98 (23.3)	276 (57.0)	112 (26.9)	105 (32.6)	127 (32.3)	73 (18.9)
Vocational school (complete)	17 (4.7)	42 (7.4)	43 (9.5)	53 (11.5)	7 (2.1)	11 (2.5)	31 (4.1)	19 (4.5)	35 (7.2)	7 (1.7)	29 (9.0)	26 (6.6)	19 (4.9)
Vocational school (incomplete)	2 (0.6)	3 (0.5)	6 (1.3)	3 (0.6)	2 (0.6)	5 (1.1)	2 (0.3)	0 (0.0)	1 (0.2)	1 (0.2)	7 (2.2)	4 (1.0)	1 (0.3)
High school (complete)	40 (11.0)	15 (2.6)	28 (6.2)	141 (30.5)	5 (1.5)	78 (17.8)	164 (22.0)	30 (7.1)	39 (8.1)	24 (5.8)	39 (12.1)	36 (9.2)	28 (7.3)
Incomplete high school or lower	8 (2.2)	4 (0.7)	4 (0.9)	18 (3.9)	1 (0.3)	15 (3.4)	118 (15.8)	6 (1.4)	5 (1.0)	4 (1.0)	7 (2.2)	24 (6.1)	5 (1.3)
Had COVID-19													
No	180 (49.6)	247 (43.6)	368 (81.2)	214 (46.3)	158 (47.3)	226 (51.6)	348 (46.6)	285 (67.9)	256 (52.9)	182 (43.6)	90 (28.0)	300 (76.3)	187 (48.4)
Most likely not	48 (13.2)	53 (9.3)	35 (7.7)	47 (10.2)	24 (7.2)	31 (7.1)	68 (9.1)	13 (3.1)	47 (9.7)	27 (6.5)	16 (5.0)	25 (6.4)	27 (7.0)
Most likely yes	25 (6.9)	78 (13.8)	6 (1.3)	73 (15.8)	55 (16.5)	59 (13.5)	181 (24.2)	25 (6.0)	46 (9.5)	56 (13.4)	68 (21.1)	7 (1.8)	59 (15.3)
Yes	110 (30.3)	189 (33.3)	44 (9.7)	128 (27.7)	97 (29.0)	122 (27.9)	150 (20.1)	97 (23.1)	135 (27.9)	152 (36.5)	148 (46.0)	61 (15.5)	113 (29.3)
Relative had COVID-19													
No	108 (29.8)	113 (19.9)	187 (41.3)	115 (24.9)	60 (18.0)	111 (25.3)	321 (43.0)	98 (23.3)	88 (18.2)	73 (17.5)	55 (17.1)	223 (56.7)	90 (23.3)
Yes	255 (70.2)	454 (80.1)	266 (58.7)	347 (75.1)	274 (82.0)	327 (74.7)	426 (57.0)	322 (76.7)	396 (81.8)	344 (82.5)	267 (82.9)	170 (43.3)	296 (76.7)
Friend had COVID-19													
No	25 (6.9)	32 (5.6)	117 (25.8)	70 (15.2)	7 (2.1)	77 (17.6)	153 (20.5)	16 (3.8)	67 (13.8)	20 (4.8)	41 (12.7)	129 (32.8)	25 (6.5)
Yes	338 (93.1)	535 (94.4)	336 (74.2)	392 (84.8)	327 (97.9)	361 (82.4)	594 (79.5)	404 (96.2)	417 (86.2)	397 (95.2)	281 (87.3)	264 (67.2)	361 (93.5)

### Instruments

#### Sociodemographic Questionnaire

An *ad hoc* sociodemographic questionnaire was constructed for this study which included sex, age, educational level, employment status, marital status, COVID-19 diagnosis (self, family and friends).

*COVID-19 Vaccine Conspiracy Beliefs Scale (COVID-VCBS)*, original Spanish title *Escala de Creencias de Conspiración de Vacunas contra la COVID-19*. This Spanish-language scale was designed based on the Vaccine Conspiracy Beliefs Scale (VCBS) (32) and assesses conspiratorial thinking about COVID-19 vaccines. The COVID-VCBS consists of 7 items, which have 7 response alternatives ranging from “strongly disagree” (1) to “strongly agree” (7). The total score of the COVID-VCBS ranges from 7 to 49, where higher values indicate a higher degree of agreement with conspiracy beliefs.

The development of the COVID-VCBS was carried out in different stages: First, the original VCBS, from which the COVID-VCBS was developed, was translated from English to Spanish using the back-translation method. For this, two independent researchers, one familiar with the subject of COVID-19 vaccination and bilingual, and the other a professional translator, translated the VCBS from English to Spanish. Then, two other independent researchers unfamiliar with the first translation (one a subject matter expert and the other a language expert) translated the Spanish version back into English. A committee, made up of the four translators and two subject matter experts, members of the research team, analyzed all the translated versions and the original version for possible inconsistencies. Based on this evaluation, a harmonized and preliminary version of the VCBS in Spanish was developed. Subsequently, the preliminary version of the VCBS was administered to 10 people to check the comprehensibility and readability of the items. A report was prepared, which included information on doubts about the interpretation of some items. The results of the pilot evaluation were reviewed by the previous expert committee. No modifications were suggested, which led to a final version of the VCBS in Spanish.

For the design of the COVID-VCBS, the items of the VCBS in Spanish were adapted to the context of the current COVID-19 pandemic. To this end, the term “COVID-19” was added to each item. Thus, for example, the item “Pharmaceutical companies hide the dangers of vaccines” was modified as “Pharmaceutical companies hide the dangers of COVID-19 vaccines.” Then, a content analysis of the COVID-VCBS was performed which included 14 expert judges (health professionals with experience in the subject of vaccination) who were contacted through their e-mails and asked to evaluate the clarity, coherence and relevance of the 7 items. The criteria were scored between 0 (not at all relevant/coherent/clear) and 3 (totally relevant/coherent/clear). The final version of the COVID-VCBS was developed from these evaluations. Table 2 presents the items of the original VCBS in English, the VCBS in Spanish and the COVID-VCBS.

**Table 2:** Original English version of the VCBS, Spanish version of the VCBS, Spanish version of the COVID-VCBS and English version of the COVID-VCBS.

**Item**	**Items from the original English version of the VCBS**	**Translation of the Items in the Spanish version of the VCBS**	**Spanish version of the COVID-VCBS**	**English versión of the COVID-VCBS**
1	Vaccine safety data is often fabricated.	La información sobre la seguridad de las vacunas a menudo se inventan.	La información sobre la seguridad de las vacunas contra la COVID-19 a menudo se inventan.	Information about the safety of COVID-19 vaccines is often fabricated.
2	Immunizing children is harmful and this fact is covered up	Vacunar a los niños es perjudicial y este hecho está ocultado	Vacunar a los niños contra la COVID-19 es perjudicial y este hecho está ocultado.	Vaccinating children against COVID-19 is harmful and this fact is covered up
3	Pharmaceutical companies cover up the dangers of vaccines.	Las empresas farmacéuticas ocultan los peligros de las vacunas	Las empresas farmacéuticas ocultan los peligros de las vacunas contra la COVID-19.	Pharmaceutical companies cover up the dangers of COVID-19 vaccines
4	People are deceived about vaccine efficacy.	Se engaña a las personas sobre la eficacia de las vacunas	Se engaña a las personas sobre la eficacia de las vacunas contra la COVID-19.	People are being misled about the efficacy of COVID-19 vaccines
5	Vaccine efficacy data is often fabricated	La información sobre la eficacia de las vacunas a menudo se inventan	La información sobre la eficacia de las vacunas contra la COVID-19 a menudo se inventan.	COVID-19 Vaccine efficacy information is often fabricated
6	People are deceived about vaccine safety.	Se engaña a las personas sobre la seguridad de las vacunas	Se engaña a las personas sobre la seguridad de las vacunas contra la COVID-19.	People are deceived about COVID-19 vaccine safety
7	The government is trying to cover up the link between vaccines and autism.	El gobierno está tratando de ocultar el vínculo entre las vacunas y la aparición de otras enfermedades	El gobierno está tratando de ocultar el vínculo entre las vacunas contra la COVID-19 y la aparición de otras enfermedades	The government is trying to cover up the link between COVID-19 vaccines and autism

*This translation followed a process of forward and back translation with a focus group to review the translated Spanish version*.

#### Single Item of Intention to Be Vaccinated Against COVID-19

A single item was developed to measure intention to be vaccinated (“How likely would it be that you decide to get vaccinated against COVID-19?” [“¿Qué tan probable sería que decidiera vacunarse contra el COVID-19?”]), which had the following response options: 1 = Not at all likely [nada probable], 2 = Not very likely [muy poco probable], 3 = Unsure [inseguro], 4 = Somewhat likely [algo probable] and 5 = Very likely [muy probable]. A higher score on the single question would express a greater intention to be vaccinated against COVID-19. During the COVID-19 pandemic, the use of single-item measures to assess intention to be vaccinated has been widespread (69, 70).

### Procedure

**Figure 1:**
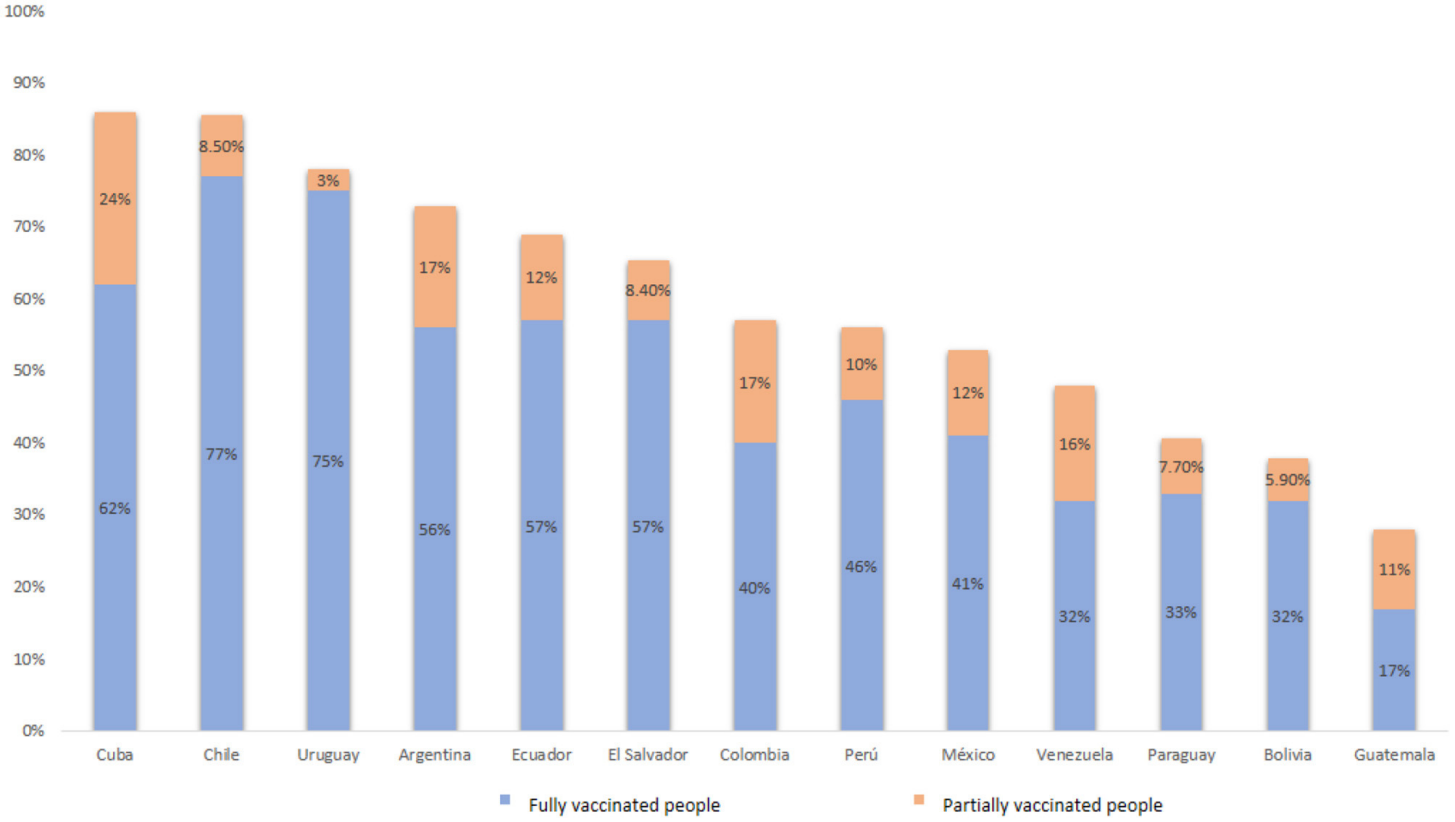
Percentage of people vaccinated against COVID-19 by October 25, 2021 in participating countries based on data derived from Our World in Data (71).

Data collection was conducted during the COVID-19 pandemic between September 15 and October 25, 2021. In that time period, between 29 and 87% of people in the countries assessed were either fully or partially vaccinated against COVID-19 (Our World in Data, 2021). In Figure 1, the countries with the highest proportion of people fully vaccinated against COVID-19 were Chile (77%) and Uruguay (75%); while Guatemala had the lowest proportion of fully vaccinated people (17%).

The data collection procedure was the same in the 13 countries participating in the study and was carried out simultaneously in all of them. For this, an online questionnaire was created with Google Forms, which was distributed through social media platforms (Facebook, Instagram and LinkedIn), personal networks, and *via* email with the detailed objectives of the study. The announcement included the Google Forms link and provided all the instructions for completing the survey. Participants were also asked to disseminate the survey link to other personal and professional contacts. Participants took part in the study voluntarily, giving informed consent after reading the study objectives. No reward or financial compensation was provided for participation. Participants were required to answer all items in order to submit their responses. The study was approved by the Ethics Committee of the Universidad Privada del Norte in Peru (registration number: 20213002).

### Data Analysis

First, the evidence of content validity of the items was evaluated from three indicators (clarity, coherence and relevance) by calculating Aiken's V and their 95% confidence intervals (95%CI). Aiken's V allows for the quantification of content validity as performed by a certain number of judges on the importance of an item with respect to a characteristic being evaluated, using the following mathematical formula, where X is the average of the judges' scores; l is the lowest score that can be obtained and k is the difference between the highest and lowest score. The score obtained ranges from 0 to 1, where values close to 1 indicate a higher degree of agreement among the judges (72):


$$\begin{array}{l} V=\frac{\bar{X}-l}{k} \end{array}$$

For the calculation of the confidence intervals (CI) the formula was used where L and U represent the lower and upper limit respectively; n is the number of judges; k is the difference between the highest and lowest score that can be obtained; V is the value of Aiken's V; and, finally, z is the standard distribution that is generally either 90, 95 or 99% (73):


$$\begin{array}{lll} L & = & \frac{2nkV+z^{2}-z\sqrt{4nkV\left(1-V\right)+z^{2}}}{2\left(nk+z^{2}\right)}\\ U & = & \frac{2nkV+z^{2}-z\sqrt{4nkV\left(1-V\right)+z^{2}}}{2\left(nk+z^{2}\right)} \end{array}$$

For the calculation of V and its CIs, the R code provided by Ventura-León (74) was followed. As mentioned before, the content analysis of the COVID-VCBS items was performed by 14 expert judges who were contacted through their e-mails and asked to evaluate the clarity, coherence and relevance of the 7 items. The criteria were scored between 0 (not relevant/coherent/clear [nada relevante/coherente/claro]) y 3 (totally relevant/coherent/clear [totalmente relevante/coherente/claro]). Values of V ≥ 0.70 and a lower limit of the 95% CI ≥ 0.59 indicate a positive evaluation of the items at the sample and population levels, respectively (75). Second, item-level descriptive statistics were examined. In addition to the mean and standard deviation, skewness and kurtosis were calculated; values between−1 and +1 were considered evidence of approximate univariate normality (76). A confirmatory factor analysis (CFA) was then performed in each country separately. The estimation method used was a robust version of maximum likelihood (MLR), which controls for non-normality of the data (77). To evaluate the fit, the following approximate indices were considered: comparative fit index (CFI), Tucker-Lewis index (TLI), root-mean-square error of approximation (RMSEA) and standardized root-mean-squared residual (SRMR). As a guide to interpretation, the following values were considered as indicators of good fit: CFI >0.95, TLI >0.95, RMSEA <0.06 and SRMR <0.08 (78).

Traditionally, measurement invariance has been examined through multigroup CFA (79). However, this procedure is not practical when there are many groups (as in the present case), so the alignment method has been proposed as an efficient alternative (54). This method starts estimating the configural model, to which an optimization algorithm is then applied to minimize the non-invariance at the level of factor loadings and intercepts. In this way, it is possible to make unbiased comparisons of the latent means. Following Fischer and Carl (80), relatively strict tolerance criteria were set for factor loadings (λ = 0.40) and intercepts (ν = 0.20). The power of alignment was set at 0.25 for both parameters. The level of invariance was evaluated with *R*^2^, one for each parameter; values close to 1 indicate high invariance. Likewise, the percentage of non-invariant parameters was examined, for which a criterion of 25% was followed to consider the scale as non-invariant (81). It should be noted that, according to psychometric theory, intercept invariance is required to make valid comparisons between groups (79). The fact that intercepts are invariant means that two people with the same level of the construct under study will tend to have the same observed score regardless of the group to which they belong (82).

After testing for approximate invariance, a graded response model (GRM) was fitted to the total sample. The GRM, which is part of the item response theory family, is a two-parameter model for polytomous items (83). These parameters are discrimination (*a*) and difficulty (*b*). One discrimination parameter is estimated for each item and its interpretation is similar to that of factor loadings in CFA. On the other hand, *k*-1 difficulty parameters (*b)* are estimated for each item, where *k* is the number of response options. Each *b* is interpreted as the level of the latent variable at which the probability of answering higher response options is 50%. The aforementioned parameters are estimated using specialized software, which applies the mathematical models of the GRM to the study data. In addition, with the estimated *a* and *b* parameters, item information curves were obtained, which show the psychometric quality of the items at different levels of the construct studied. The concept of information in the IRT is similar to the more traditional concept of reliability, so the information curves can be interpreted as a graphical representation of the reliability of the items in people with different degrees of the variable under study, which in this case is conspiratorial beliefs about vaccination (82). These information functions are obtained from specialized formulas, as described in Fraley et al. (84).

Subsequently, we sought evidence of validity based on relationships with other variables. For this purpose, the association between the COVID-VCBS and a single item of intention to be vaccinated was examined. To control for possible sources of measurement error, this analysis was performed with a CFA. In order to construct a latent variable with the single item of intention to be vaccinated, the procedure described by Brown (85) was followed, setting the reliability of the single indicator as 0.80 according to the recommendation of Savalei (86).

Finally, comparisons were made between observed COVID-VCBS scores across countries. Because large sample sizes could lead to statistically significant results even in the absence of practical significance, an approach based on effect size was used. Specifically, Cohen's d was calculated between all country pairs and boxplots were used for a visual inspection of the differences. As a guide for interpretation, values of 0.20, 0.50 and 0.80 were considered as small, medium and large differences, respectively (87).

All analyses were performed in R, version 4.0.3. In addition, the following packages were used: lavaan 0.6–8 (for the single-group CFAs), sirt 3.9–4 (for the approximate invariance analysis), mirt 1.33.2 (for the GRM), rstatix 0.6.0 (for Cohen's d) and ggpubr 0.4.0 (for the boxplots).

## Results

### Preliminary Analyses

First, Table 3 shows that all of the items in the COVID-VCBS present V values ≥ 0.79 and lower limits of CIs ≥ 0.59. In this sense, all items are relevant (i.e., important and should be included in the measurement of conspiracy beliefs), representative of the construct they are intended to measure, as well as clear and understandable. Table 4 shows the descriptive statistics at the item level. As can be seen, in almost all countries item 1 (“COVID-19 vaccines safety data are often fabricated”) showed the largest mean. Likewise, in all cases the item means were around 3 or 4.5, with variations between countries. It is also observed that in most cases the skewness and kurtosis values were within the range between −1 and +1, suggesting approximately normal univariate distributions.

**Table 3:** Aiken's V for assessing the clarity, consistency and relevance of the COVID-VCBS items.

**Item**	**Relevance**				**Coherence**				**Clarity**			
	**M**	**SD**	**V**	**CI95%**	**M**	**SD**	**V**	**CI 95%**	**M**	**SD**	**V**	**CI 95%**
1	2.71	0.47	0.90	0.75–0.97	2.57	0.65	0.86	0.69–0.94	2.71	0.47	0.90	0.75–0.97
2	2.50	0.52	0.83	0.66–0.93	2.21	0.58	0.74	0.59–0.86	2.21	0.43	0.74	0.56–0.86
3	2.64	0.74	0.88	0.72–0.96	2.71	0.73	0.90	0.75–0.97	2.64	0.74	0.88	0.72–0.96
4	2.36	0.63	0.79	0.61–0.90	2.43	0.51	0.81	0.64–0.91	2.50	0.52	0.83	0.66–0.93
5	2.86	0.36	0.95	0.81–0.99	2.71	0.61	0.90	0.75–0.97	2.50	0.76	0.83	0.66–0.93
6	2.57	0.51	0.86	0.69–0.94	2.79	0.43	0.93	0.78–0.98	2.79	0.43	0.93	0.78–0.98
7	2.36	0.63	0.79	0.61–0.90	2.21	0.58	0.74	0.56–0.86	2.36	0.63	0.79	0.61–0.90

*M, mean; SD, standard deviation; V, Aiken's V; CI, Confidence Interval*.

**Table 4:** Item-level descriptive statistics of the COVID-VCBS items.

**Country**	**Statistic**	**COVID-VCBS Items**						
		**1**	**2**	**3**	**4**	**5**	**6**	**7**
Argentina (*n* = 363)	*M*	3.69	2.47	3.24	2.96	3.07	2.98	2.84
	*SD*	1.88	1.70	1.77	1.68	1.74	1.65	1.71
	*g1*	0.07	1.02	0.40	0.54	0.44	0.52	0.60
	*g_2_*	−0.98	0.27	−0.70	−0.53	−0.77	−0.47	−0.45
Bolivia (*n* = 567)	*M*	3.73	3.25	3.88	3.78	3.56	3.59	3.56
	*SD*	1.73	1.74	1.79	1.81	1.72	1.71	1.77
	*g1*	0.10	0.37	0.06	0.15	0.26	0.23	0.18
	*g_2_*	−0.78	−0.62	−0.76	−0.87	−0.68	−0.70	−0.79
Chile (*n* = 453)	*M*	3.15	2.60	3.31	2.90	2.77	2.91	3.11
	*SD*	1.94	1.74	1.81	1.78	1.71	1.80	1.90
	*g1*	0.48	0.95	0.44	0.68	0.79	0.67	0.58
	*g_2_*	−0.94	0.07	−0.70	−0.52	−0.29	−0.55	−0.76
Colombia (*n* = 462)	*M*	3.89	2.92	3.58	3.53	3.50	3.48	3.70
	*SD*	1.83	1.65	1.76	1.83	1.73	1.76	1.86
	*g1*	−0.05	0.47	0.15	0.23	0.20	0.24	0.13
	*g_2_*	−0.79	−0.50	−0.78	−0.87	−0.75	−0.77	−0.92
Cuba (*n* = 334)	*M*	3.25	1.94	2.91	2.90	2.95	2.74	2.65
	*SD*	1.87	1.43	1.73	1.81	1.78	1.68	1.70
	*g1*	0.29	1.55	0.54	0.61	0.57	0.69	0.83
	*g_2_*	−1.00	1.96	−0.58	−0.68	−0.70	−0.51	−0.09
Ecuador (*n* = 438)	*M*	4.05	3.20	3.93	3.66	3.66	3.63	3.72
	*SD*	1.90	1.87	1.90	1.93	1.89	1.91	1.86
	*g1*	−0.08	0.45	0.06	0.16	0.19	0.25	0.13
	*g_2_*	−0.95	−0.79	−0.96	−1.06	−0.94	−0.91	−0.89
El Salvador (*n* = 747)	*M*	4.14	3.72	4.07	3.82	3.87	3.86	4.00
	*SD*	1.88	1.87	1.76	1.83	1.78	1.80	1.87
	*g1*	−0.16	0.05	−0.09	0.05	−0.02	−0.01	−0.07
	*g_2_*	−0.89	−0.92	−0.72	−0.85	−0.76	−0.80	−0.90
Guatemala (*n* = 420)	*M*	4.15	3.07	3.88	3.54	3.73	3.62	3.53
	*SD*	1.91	1.83	1.86	1.86	1.87	1.83	1.86
	*g1*	−0.19	0.44	0.06	0.21	0.16	0.22	0.31
	*g_2_*	−1.01	−0.75	−0.90	−0.92	−0.92	−0.83	−0.80
Mexico (*n* = 484)	*M*	3.64	2.67	3.09	2.95	2.98	2.88	3.11
	*SD*	1.90	1.67	1.72	1.80	1.73	1.69	1.78
	*g1*	0.12	0.67	0.41	0.55	0.49	0.53	0.33
	*g_2_*	−0.99	−0.39	−0.64	−0.64	−0.64	−0.54	−0.89
Paraguay (*n* = 417)	*M*	3.70	2.74	3.21	3.06	3.16	3.12	3.28
	*SD*	1.97	1.67	1.73	1.74	1.75	1.74	1.72
	*g1*	0.16	0.74	0.40	0.60	0.48	0.57	0.30
	*g_2_*	−1.10	−0.12	−0.61	−0.40	−0.55	−0.38	−0.58
Peru (*n* = 322)	*M*	4.16	3.46	3.99	3.93	3.93	3.98	3.88
	*SD*	1.82	1.75	1.73	1.81	1.69	1.72	1.76
	*g1*	−0.20	0.15	−0.13	−0.09	−0.18	−0.18	−0.09
	*g_2_*	−0.78	−0.71	−0.72	−0.83	−0.70	−0.72	−0.74
Uruguay (*n* = 393)	*M*	3.93	3.49	4.03	3.66	3.54	3.70	3.55
	*SD*	1.92	1.76	1.83	1.87	1.83	1.91	1.86
	*g1*	0.09	0.29	0.03	0.25	0.30	0.20	0.20
	*g_2_*	−0.98	−0.58	−0.84	−0.88	−0.79	−0.99	−0.90
Venezuela (*n* = 386)	*M*	3.59	3.33	3.76	3.32	3.37	3.37	3.74
	*SD*	1.98	1.91	1.94	1.88	1.90	1.93	1.95
	*g1*	0.23	0.37	0.16	0.47	0.38	0.42	0.18
	*g_2_*	−1.10	−0.87	−1.07	−0.79	−0.91	−0.89	−1.03
Overall (*n* = 5,786)	*M*	3.79	3.04	3.64	3.42	3.42	3.40	3.48
	*SD*	1.91	1.81	1.83	1.86	1.81	1.82	1.86
	*g1*	0.05	0.51	0.17	0.31	0.29	0.31	0.25
	*g_2_*	−1.00	−0.65	−0.85	−0.87	−0.83	−0.81	−0.88

*M, Mean; SD, Standard Deviation; g1, Skewness; g2, Kurtosis*.

A CFA was then performed for each country. As shown in Table 5, the fit was acceptable in each country separately, as well as in the total sample. In all countries, with the exception of Cuba, the lowest factor loading was for item 1. As for reliability, there was generally consistency between the alpha and omega coefficients; the values ranged between 0.87 and 0.94.

**Table 5:** Single-group confirmatory factor analyses and internal consistency reliability of the COVID-VCBS.

**Country**	**Fit indices**							**Factor loadings**							**α**	**ω**
	**χ** ^2^	**df**	**p**	**CFI**	**TLI**	**RMSEA**	**SRMR**	**1**	**2**	**3**	**4**	**5**	**6**	**7**		
Argentina	30.48	14	0.007	0.98	0.97	0.06	0.03	0.49	0.61	0.73	0.88	0.86	0.90	0.70	0.89	0.89
Bolivia	48.40	14	<0.001	0.97	0.95	0.07	0.03	0.51	0.55	0.74	0.87	0.84	0.88	0.70	0.89	0.89
Chile	40.83	14	<0.001	0.98	0.97	0.07	0.02	0.62	0.72	0.82	0.89	0.89	0.93	0.80	0.93	0.93
Colombia	54.26	14	<0.001	0.96	0.94	0.08	0.04	0.52	0.59	0.78	0.87	0.86	0.91	0.78	0.91	0.91
Cuba	17.55	14	0.228	1.00	0.99	0.03	0.02	0.70	0.58	0.81	0.91	0.90	0.92	0.75	0.92	0.93
Ecuador	47.13	14	<0.001	0.96	0.95	0.07	0.03	0.46	0.69	0.80	0.86	0.80	0.86	0.87	0.91	0.91
El Salvador	60.23	14	<0.001	0.96	0.95	0.07	0.03	0.44	0.55	0.69	0.84	0.82	0.86	0.71	0.87	0.87
Guatemala	53.16	14	<0.001	0.95	0.93	0.08	0.03	0.41	0.58	0.73	0.86	0.79	0.87	0.72	0.87	0.88
Mexico	41.47	14	<0.001	0.97	0.96	0.06	0.03	0.45	0.61	0.77	0.87	0.84	0.89	0.78	0.89	0.90
Paraguay	15.25	14	0.361	1.00	1.00	0.01	0.02	0.46	0.66	0.78	0.84	0.81	0.85	0.76	0.89	0.89
Peru	41.12	14	<0.001	0.95	0.93	0.08	0.04	0.51	0.66	0.70	0.78	0.86	0.82	0.77	0.89	0.89
Uruguay	36.71	14	0.001	0.98	0.97	0.06	0.02	0.63	0.78	0.86	0.91	0.90	0.92	0.81	0.94	0.94
Venezuela	43.91	14	<0.001	0.97	0.95	0.07	0.03	0.49	0.63	0.78	0.87	0.90	0.91	0.66	0.90	0.90
Overall	339.68	14	<0.001	0.97	0.96	0.06	0.02	0.52	0.64	0.77	0.87	0.85	0.89	0.76	0.90	0.91

### Approximate Measurement Invariance

When examining the approximate invariance with the alignment method, it was observed that the COVID-VCBS is invariant for both factor loadings (R^2^ = 0.996) and intercepts (R^2^ = 0.998). The first block of Table 6 corresponds to the invariance of factor loadings; as can be seen, all of them were invariant between countries (0% of non-invariant parameters). On the other hand, in the second block, referring to the invariance of intercepts, some invariant parameters are observed, which is indicated with parentheses around the abbreviation of the countries. However, this value (15.4%) was below the pre-established criterion of 25%, which leads to the conclusion that the intercepts show approximate invariance. The latent means (which take Argentina as the reference group) are also presented. As can be seen, Cuba obtained the lowest mean, while the highest ones corresponded to Peru and El Salvador.

**Table 6:** Approximate measurement invariance of the COVID-VCBS using the alignment method.

**Parameters**	**Items**	**Countries**													***R^2^***	**%**
Factor loadings	1	AR	BO	CL	CO	CU	EC	SV	GT	MX	PY	PE	UY	VE	0.996	0.0
	2	AR	BO	CL	CO	CU	EC	SV	GT	MX	PY	PE	UY	VE		
	3	AR	BO	CL	CO	CU	EC	SV	GT	MX	PY	PE	UY	VE		
	4	AR	BO	CL	CO	CU	EC	SV	GT	MX	PY	PE	UY	VE		
	5	AR	BO	CL	CO	CU	EC	SV	GT	MX	PY	PE	UY	VE		
	6	AR	BO	CL	CO	CU	EC	SV	GT	MX	PY	PE	UY	VE		
	7	AR	BO	CL	CO	CU	EC	SV	GT	MX	PY	PE	UY	VE		
Intercepts	1	AR	BO	(CL)	CO	CU	EC	SV	(GT)	MX	PY	PE	(UY)	(VE)	0.998	15.4
	2	AR	(BO)	CL	CO	(CU)	EC	(SV)	GT	MX	PY	PE	UY	(VE)		
	3	AR	BO	CL	CO	CU	EC	SV	GT	MX	PY	PE	UY	VE		
	4	AR	(BO)	CL	CO	CU	EC	SV	GT	MX	PY	PE	UY	VE		
	5	AR	BO	CL	CO	CU	EC	SV	GT	MX	PY	PE	(UY)	VE		
	6	AR	BO	CL	CO	CU	EC	SV	GT	MX	PY	PE	UY	VE		
	7	(AR)	BO	CL	CO	(CU)	EC	SV	GT	MX	PY	PE	(UY)	(VE)		
Factor means		0.00	0.38	−0.05	0.32	−0.15	0.42	0.56	0.41	−0.05	0.07	0.62	0.46	0.25		

*Non-invariant parameters are presented in parentheses. The percentage of non-invariant parameters in presented in the last column. AR, Argentina; BO, Bolivia; CL, Chile; CO, Colombia; CU, Cuba; EC, Ecuador; SV, El Salvador; GT, Guatemala; MX, Mexico; PY, Paraguay; PE, Peru; UY, Uruguay; VE, Venezuela*.

### Graded Response Model

Table 7 presents the parameters estimated with the GRM. As can be seen, the lowest discrimination parameter (*a*) was that of item 1, while the highest corresponded to item 6. This means that item 6 best distinguishes between people with low and high levels of conspiracy beliefs about the vaccine. For the difficulty parameters (*b*), it can be seen that item 1 required more extreme levels of the construct to select the first or last options. As for item 2, this was only observed for the last response option (i.e., a level of conspiracy beliefs more than 2 *SD* above the mean was required to have a 50% probability of selecting the last response option). For the remaining items, the difficulty parameters were very similar.

**Table 7:** Estimated parameters of the graded response model applied to the COVID-VCBS.

**Item**	***a***	***b1***	***b_2_***	***b3***	***b4***	***b5***	***b6***
1	1.31	−1.44	−0.80	−0.27	0.75	1.38	2.02
2	1.80	−0.68	−0.23	0.23	1.25	1.65	2.13
3	2.67	−1.09	−0.59	−0.59	0.66	1.09	1.54
4	4.19	−0.83	−0.36	−0.36	0.70	1.05	1.46
5	3.94	−0.88	−0.38	−0.38	0.75	1.11	1.56
6	4.98	−0.83	−0.36	−0.36	0.72	1.08	1.46
7	2.59	−0.93	−0.46	−0.46	0.76	1.17	1.61

*Parameter a is the discrimination parameter, while the b parameters are difficulty parameters from item response theory*.

**Figure 2:**
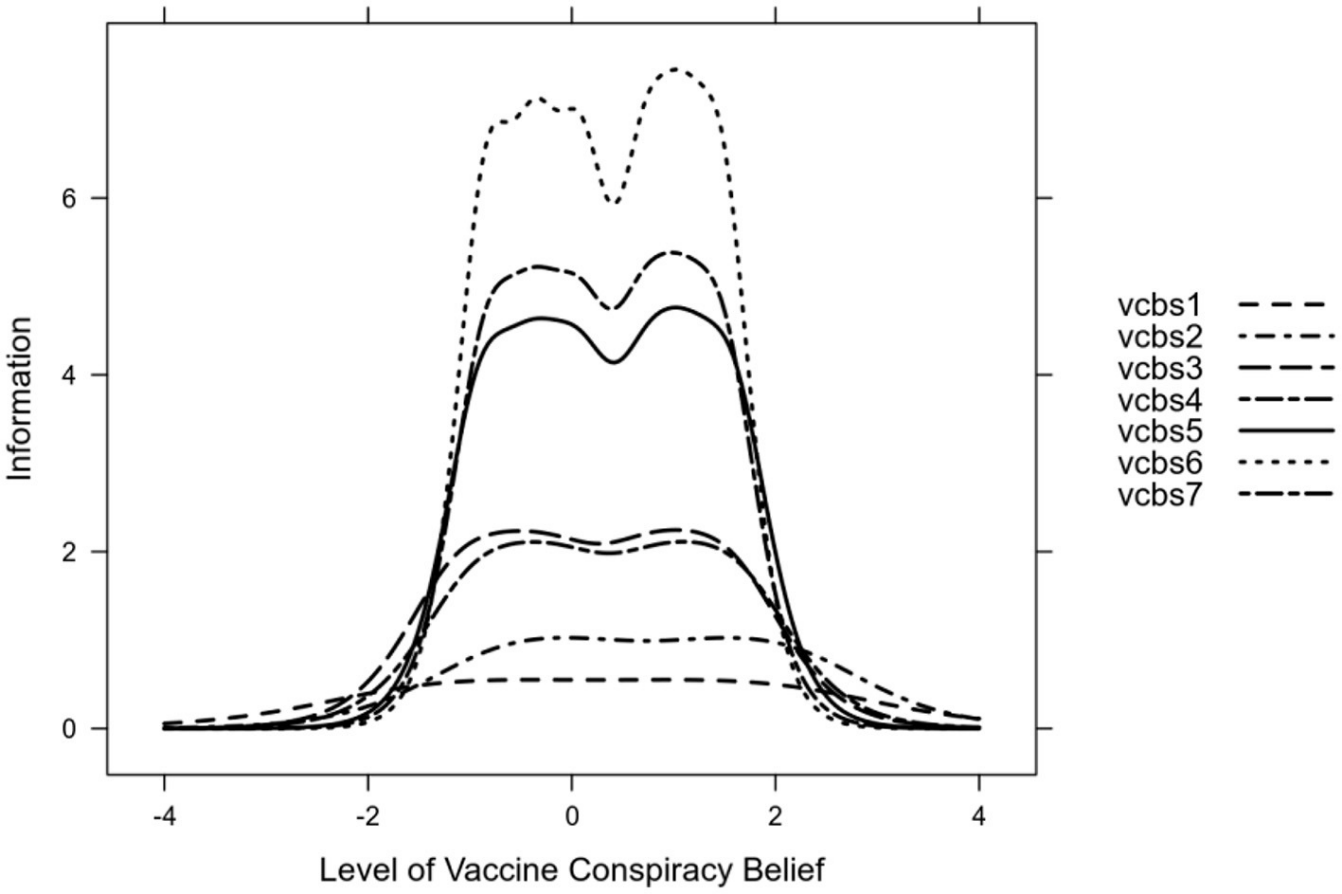
Item information curves of the COVID-VCBS.

Using the GRM parameters, a series of item information curves was constructed (Figure 2). These confirm that item 6 is the most informative, while the least informative is item 1. Also, the scale seems to be most informative (reliable) between ~ −1.5 and +2 *SD*.

### Associative Validity

Based on a CFA, the COVID-VCBS total score showed a medium correlation with the intention to be vaccinated, ϕ = −0.34, 95% CI: [−0.37, −0.31], *p* < 0.001. It is worth noting that this measurement model obtained an adequate adjustment, CFI = 0.97, TLI = 0.96, RMSEA = 0.06, SRMR = 0.03.

### Mean Comparison Across Countries

**Figure 3:**
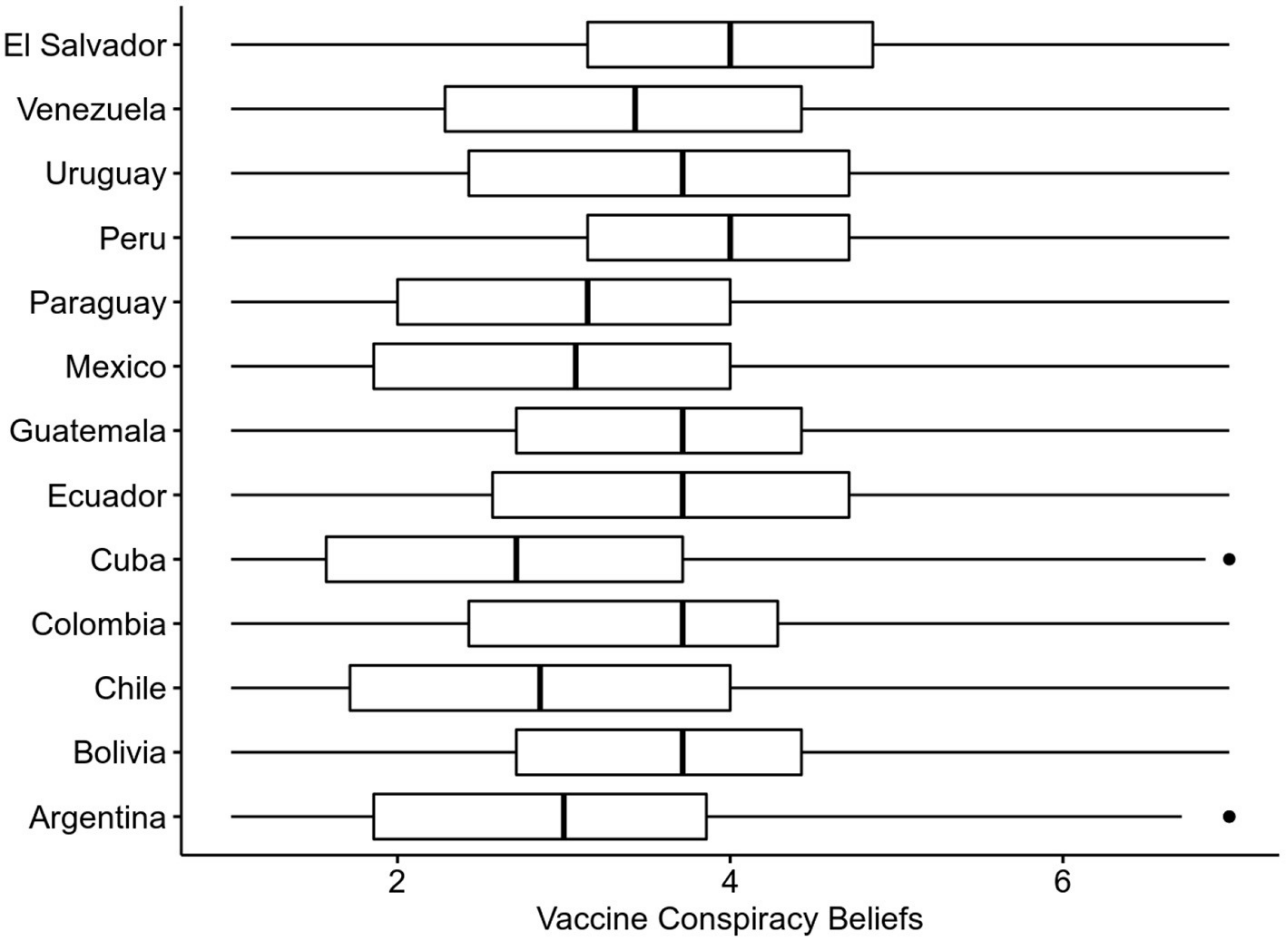
Boxplots comparing the observed scores of the COVID-VCBS across countries.

To complement the comparison of latent means made with the alignment method, a comparison of observed means between countries was also made. When comparing these, small and negligible differences were observed in most cases. The largest difference occurred between the scores of Cuba and El Salvador (*d* = 0.83). In addition to this large difference, some medium differences were also observed (the country with the lower mean is mentioned first): between Argentina and Peru (*d* = 0.64), between Argentina and El Salvador (*d* = 0.66), between Cuba and Bolivia (*d* = 0.62), between Chile and Peru (*d* = 0.65), between Chile and El Salvador (*d* = 0.67), between Cuba and Colombia (*d* = 0.53), between Cuba and Ecuador (*d* = 0.63), between Cuba and Guatemala (*d* = 0.62), between Cuba and Uruguay (*d* = 0.62), between Mexico and Peru (*d* = 0.63), between Mexico and El Salvador (*d* = 0.64), between Paraguay and Peru (*d* = 0.53), and between Paraguay and El Salvador (*d* = 0.54). Figure 3 presents a graphical representation of the differences between countries. It can be observed that Peru was one of the countries with the highest level of conspiracy beliefs about the vaccine, while the countries with the lowest average were Cuba and Chile.

## Discussion

For some years now, there has been a growing interest in conducting research across cultures and/or countries (88). However, conducting this type of study involves several challenges. One of the main ones is related to the instruments used and, specifically, to the limited number of studies that assess the MI of a construct across countries (89). The assessment and investigation of conspiracy beliefs about COVID-19 vaccines is a particularly relevant issue during the current pandemic. Even more so if there is a group of anti-vaccine individuals who justify their behavior on conspiratorial views and without demonstrating fear of the consequences of COVID-19 (87). Therefore, the main objective of the present study was to develop and evaluate the cross-cultural MI of the COVID-19 Vaccine Conspiracy Beliefs Scale (COVID-VCBS) in 13 Latin American countries.

First, using the CFA, the unidimensional structure of the COVID-VCBS in the 13 participating countries was confirmed. This is similar to that reported in the VCBS from which the COVID-VCBS is derived (32). The finding is consistent with the assumption that conspiracy mentality is, in essence, a unidimensional construct (29). Unidimensionality is a basic assumption for validly calculating total scores, whether from CTT more modern theories such as IRT (38, 90). Previously, it has been suggested that it is sufficient to obtain a total score that measures people's tendency to engage in conspiratorial ideas (29). In this sense, the items of the COVID-VCBS represent a common underlying variable (or latent variable) and that allows for obtaining an interpretable total score across countries (91). Likewise, it is suggested that scores representing a single defined attribute are needed to have an unambiguous interpretation, and these scores should not be significantly influenced by other variables. If multiple attributes are measured, it will not be clear which of them should be used to report a particular score (92). Likewise, the results confirm the adequate reliability of the COVID-VCBS in all countries, with alpha and omega coefficient values above 0.87. That is, the COVID-VCBS appears to provide accurate scores in all countries participating in the study.

In addition, the present study tested the MI of the COVID-VCBS in 13 countries. The approximate invariance results, using the alignment method, confirmed the MI of the COVID-VCBS for both factor loadings and intercepts. This suggests that the COVID-VCBS is assessing the same underlying construct of conspiracy beliefs about the COVID-19 vaccine at the level of the 13 Latin American countries. To the best of our knowledge from the scientific literature, this is the first study to test the MI of a measure of conspiracy beliefs about COVID-19 vaccines across different countries. This type of finding helps to avoid inference problems when comparing results from different countries and, therefore, allows for more rigorous and robust conclusions to be drawn in cross-cultural research on conspiracy beliefs. Furthermore, this study is the first to employ the alignment method to assess the MI of a conspiracy belief scale. This is a novel statistical approach to test MI in a large number of groups, in this case, 13 Latin American countries. As expected, the factor loadings of the items demonstrated a greater amount of invariance compared to the crosstabs (93). Specifically, items 3, 4 and 6 were the most invariant and dealt with the efficacy and safety of COVID-19 vaccines. Currently, beliefs about vaccine safety and efficacy are important predictors of the decision to be vaccinated against COVID-19 in the global population (94). This should lead policymakers to build confidence in the safety and efficacy of COVID-19 vaccines offered to the general population in Latin American countries. Given that there is still uncertainty about the risks of vaccines against COVID-19, information without scientific evidence provided by some media about vaccines can cause fear in people and lead to refusal of vaccination. In this regard, there is a need to educate the media and the general public that information without scientific evidence is not a valid way to determine the safety or efficacy of COVID-19 vaccines. The use of the alignment method allows for MI testing and comparison of latent means in large-scale data, something that is difficult to achieve with the MGCFA method. Handling data in a large number of groups, allows for testing for invariance in different subpopulations within countries and cohorts (95). Estimation and comparison of latent means can be performed even though the measures are not fully or partially invariant (96). Thus, the alignment method significantly simplifies comparative analyses, but does not prevent it from producing unbiased statistical estimates of group means, with significance tests adjusting for sampling errors and missing data (95).

Once the MI was tested, the analysis of the discrimination and difficulty parameters, using IRT, provides further information on the performance of the COVID-VCBS items. Item 6 (“People are misled about the safety of COVID-19 vaccines”) presented the highest discrimination parameter, indicating that this item can generate very varied responses, ranging from strongly disagree to strongly agree, in people with different levels of conspiracy beliefs about COVID-19 vaccines. That is, item 6 would better distinguish between people with different levels of conspiracy beliefs about vaccines. People's responses to item 6 of the COVID-VCBS would provide more information about conspiracy beliefs because it is referred to having transparent information about vaccine safety, which is one of the most influential factors for vaccination (97). Specifically, the belief in safety regarding the COVID-19 vaccine is, together with efficacy and effectiveness, the factor that underpins the strategy of any vaccination program and its acceptance by the population (98). However, there is regional variability in the perception of vaccine safety that needs to be considered. For example, the majority of low-income people in South Asia (95%) and East Africa (92%) agreed that vaccines are safe; while these rates were lower in people with higher incomes in North America (72%), Northern Europe (73%), Western Europe (59 %) and Eastern Europe (50 %) (10). In this sense, it seems that the level of economic income significantly mediates these differences. The results of the difficulty parameters and the item response curves suggest that item 6 is the most informative, important and provides the greatest robustness to the COVID-VCBS. People who are more accepting of conspiracy beliefs about vaccines will find it easy to respond to item 6 compared to those who do not tend to accept these beliefs. Similarly, the difficulty parameters of the items are always ascending. This indicates that a higher presence of the latent trait, i.e., a higher acceptance of vaccine conspiracy beliefs, is needed to respond to the higher response categories.

Additionally, the presence of MI allowed us to explore differences in the means of conspiracy beliefs about COVID-19 vaccines across the 13 countries. Two main patterns of results emerged when analyzing the differences between countries. First, Cuba and Chile obtained the lowest means; while Peru presented the highest means. It has been suggested that the existing differences can be explained by trust toward vaccination (99), where lower levels of general trust predict higher acceptance of conspiracy theories, which is in agreement with previous findings (100). In this sense, in order to foster trust about vaccines, not only the content of the messages about vaccination is important, but also the medium from which they come from. Thus, it is suggested that people have more trust and adapt to the behaviors of those closest to them; therefore, in minority groups, information about vaccines coming from a family member may be more effective than information coming from an outsider (101). In the Cuban case, a previous study reported that they have the highest levels of COVID-19 information and satisfaction and trust with the information provided by experts in their country (102). This would suggest that, in Cuba, trust in information provided by the government affects the behavior of COVID-19 vaccine safety. In contrast to Cuba, in the UK, people who rely on celebrities for information about different aspects of COVID-19 are more resistant to disinformation about COVID-19; whereas, in the US, people who relied on family or friends for information are less resistant to disinformation (14). In China, more than 85.0% of people indicated all sources of information about the COVID-19 vaccine are good/excellent (103).

In the case of Chile, the low acceptance of conspiracy beliefs about COVID-19 vaccines was related to the fact that only 23% of the population refused to be vaccinated at all (104). Chile, together with Brazil, had the highest acceptance rates compared to other Latin American countries (105), which were even higher than other countries in Europe, Asia and Africa (10). On the other hand, in Peru, the lack of trust in scientific information about COVID-19 in general and vaccines in particular has led to conspiracy ideas being present in numerous scenarios. Thus, for example, in the town of Huancavelica, a group of fanatical people kidnapped workers who were doing maintenance on cell phone antennas with 5G technology, thinking that these were spreading the SARS-CoV-2 infection (106). Additionally, Peruvian congressmen requested the creation of a commission to evaluate the effects of chlorine dioxide in the treatment of COVID-19, and invited advocates of chlorine dioxide to present their ideas (107). This is similar to what is happening in other countries in Europe, such as Croatia and Slovenia, where lack of trust in science and scientists, as well as political impotence were also the strongest predictors of acceptance of conspiracy beliefs during the pandemic (108, 109). Similarly, in Asian countries, people who had scientists and scientific journals as their main source of information about COVID-19 vaccines were less likely to believe in conspiracies about vaccines (15). While this was not an overall objective, the findings comparing conspiracy beliefs across the 13 countries allow us to emphasize the importance of assessing the relationship between cultural factors and information consumption as expressed in conspiracy theories at a time of crisis, such as the COVID-19 pandemic (110).

Finally, there is evidence on the relationship between beliefs in conspiracies about vaccination and health-related behaviors (63). Thus, this study showed that higher conspiracy beliefs about vaccination against COVID-19 were associated with lower vaccination intention in the 13 countries of Latin America and the Caribbean. This is in line with previous studies that reported a negative relationship between these variables in different countries in North America, Asia, Europa and elsewhere (25, 63–65, 111). Although the correlation between the variables is moderate, the unprecedented scale of the current pandemic makes this relationship very dangerous, to the extent that any reluctance to be vaccinated could represent a major threat to public health (112). On the other hand, the correlation between the variables in this study is slightly stronger than reported by previous studies in the United States and France, where the effect sizes of the correlations ranged from r2 = 0.05 (25) to r2 = 0.27 (113). This might suggest that the negative relationship between conspiracy beliefs about COVID-19 vaccines and intentions to be vaccinated against the disease appears to strengthen as the disease has progressed. However, this does not mean that conspiracy beliefs became stronger over time; rather, as the pandemic has progressed, conspiracy beliefs about COVID-19 vaccines have become an important factor in distinguishing those most likely to be vaccinated. Unlike the vast majority of studies, this study was conducted when COVID-19 vaccination programs were underway in all of the Latin American countries evaluated. Therefore, participants did not provide information about their beliefs in a future scenario, since the COVID-19 vaccine was available to them. It has been suggested that this situation may have generated more accurate responses because participants did not have to imagine a hypothetical situation (114).

It has been suggested that the relationship between conspiracy beliefs about COVID-19 vaccines and intention to be vaccinated against COVID-19 may be mediated by perceived risk of vaccines, feelings of helplessness and distrust of authorities (63). This relationship can also be explained by a cost-benefit analysis (17). Thus, the perceived dangers associated with conspiracy beliefs about vaccines may outweigh the perceived benefits and reduce the intention to be vaccinated (31). Likewise, the relationship between conspiracy beliefs about COVID-19 vaccines and intention to be vaccinated can also likely be explained by a general psychological tendency to believe in conspiracies (113). In this sense, previous studies have suggested that the acceptance of traditional conspiracy beliefs, such as those referring to the arrival of people on the moon, are associated with negative attitudes toward vaccines (113); while both conspiratorial beliefs about COVID-19 and having a conspiratorial mindset in general negatively predicted intentions to be vaccinated against COVID-19 (25). However, the latter is an issue that deserves further investigation, as a previous study suggested that only belief in vaccine-related conspiracy theories had a significant negative impact on intentions to be vaccinated, while belief in pandemic conspiracy theories in general did not have a significant impact on intentions to be vaccinated (115).

This finding leads to suggest that countering conspiracy beliefs about COVID-19 vaccines should play an important role in actions aimed at increasing intentions to be vaccinated against COVID-19 in the 13 countries evaluated. Intervening against conspiracy beliefs involves having a comprehensive approach where messages are clear and based on scientific evidence, as well as delivered through legitimate channels. The mitigation of misinformation should result from the collaboration of the scientific community and the media, which should verify facts and call out content that disseminates misinformation. On the other hand, while the findings make it clear that conspiracy beliefs about COVID-19 vaccines play a role in COVID-19 vaccination intention, little is yet known about the etiology of the beliefs. In this regard, the use of qualitative or mixed methods in future studies could provide more information about the origins of these conspiracy beliefs and help design effective interventions to increase COVID-19 vaccine acceptance.

The study also has some limitations that should be considered when interpreting the results. First, the generation of the COVID-VCBS items was deductive rather than inductive; that is, the items were based on already existing knowledge (VCBS items). In this sense, it is possible that some aspects of pandemic-specific conspiracy beliefs were overlooked and/or the existing items might not optimally capture this construct. Similarly, self-report measures are inevitably affected by measurement error and social desirability biases. Therefore, it would be beneficial for future studies to use triangulation methods, such as assessing the convergence of COVID-VCBS scores with data reported from other sources, such as, for example, interviews and/or information obtained from close associates. Second, in all countries, data were collected by convenience sampling. Therefore, the samples used in each participating country are not fully representative of the entire population due to different aspects. For example, the recruitment of participants through social networks, such as Facebook and Instagram, did not reach those people who do not use these websites, nor did it reach people who do not use the Internet. Similarly, comparisons of the COVID-VCBS scores may be somewhat biased because some characteristics among the 13 countries were not directly comparable. Thus, although the study included participants of different sexes, women represented more than 70% of the total sample. There is evidence of gender differences regarding conspiracy beliefs about COVID-19 vaccines, where women are more supportive of conspiracy beliefs against a vaccine (15, 21). Future studies could investigate the invariance of the COVID-VCBS between sexes within each of the countries to assess whether men and women respond to the items differently. In addition, most of the participants were university educated, and participants with less education would probably have reduced access to the Internet. Additionally, the Mexican sample were the youngest, with an average age of 25 years; while the Guatemalans had an average age of 44 years. With an age difference of almost 20 years, the comparison of COVID-VCBS scores between Mexicans and Guatemalans could obviously be biased by age. Future studies are encouraged to improve the sampling design, for example, by using stratified sampling. This would allow for samples in each country to be more representative and lead to more generalizable results.

Third, the study samples comprise 13 countries. This implies that, the findings related to the COVID-VCBS MI only apply to these countries. Fourth, the study data were collected between September 15 and October 25, 2021, several months after the development and distribution of different highly effective COVID-19 vaccines. This circumstance probably influences the hesitations experienced by the participants regarding the vaccine at the time of the evaluation and could have affected some findings, such as the estimates of item difficulty. Fifth, the cross-sectional study design, which did not allow us to assess the direction of causality between conspiracy beliefs and fear of COVID-19 or to test longitudinal MI. There is evidence suggesting the presence of changes in conspiracy beliefs about COVID-19 vaccines (116); thus, future studies should assess the temporal stability of the COVID-VCBS. Sixth, while establishing measurement invariance among 13 different countries is important for generalizability of the COVID-VCBS, future studies need to include an English or other language version of the scale for additionally cross-cultural comparability. Seventh, an important area of research involves the factors that contribute to the emergence of conspiracy beliefs about COVID-19 vaccines and their relationships to other variables. For example, it has been suggested that higher levels of trust in health care institutions are associated with lower support for conspiracy theories and, therefore, greater willingness to be vaccinated against COVID-19 (117–120). However, it has also been suggested that citizens' trust in institutions may generally influence whether or not they support conspiracy theories, but that they do not determine whether beliefs translate into prevention behaviors (112). Other variables must also be considered in order to understand vaccine conspiracy theories in Latin America, which have been shown to play an important role in other contexts such as the United States, Europe and Asia, such as trust in science, political orientation and populism (121, 122), anomie and health threat perceptions (123) and vaccine literacy (115), among others. In Latin America, it has been suggested that ethnic discrimination (62), sex, age, the level of education and the medium through which information about the COVID-19 vaccine is received (124) are variables related to the acceptance or non-acceptance of conspiratorial ideas about the COVID-19 vaccines. To this end, future studies should incorporate predictive designs that allow for a better understanding of the relationships between these and other variables.

Despite these limitations, this study's results may offer important contributions to the investigation of conspiracy beliefs about COVID-19 vaccines. By providing evidence of validity and cross-cultural MI of the COVID-VCBS to assess conspiracy beliefs about COVID-19 vaccines in adults from 13 different countries, the study opens new avenues for the assessment of conspiracy beliefs about vaccines internationally. In this sense, it contributes to the body of cross-cultural studies by establishing the possibility of cultural comparison of conspiracy beliefs about COVID-19 vaccines using the same measurement instrument. The COVID-VCBS is practical, brief, and easy to apply; thus, having an instrument with reduced cultural influence is useful for comparing data and obtaining information on similarities and differences between different countries regarding their vaccine conspiracy beliefs. In addition, understanding cross-cultural differences can be expanded by identifying non-invariant items and countries using the alignment method. Understanding non-invariance will allow for the development of more culturally invariant items in the future. As we know, the presence of conspiracy beliefs interferes with adherence to preventive behaviors against COVID-19 (22). Thus, being able to measure conspiracy beliefs about COVID-19 vaccines among Latin American countries will allow us to gain a better understanding of this construct that is so important for the success of COVID-19 vaccination programs in this region.

In conclusion, the results revealed that the COVID-19 Vaccine Conspiracy Beliefs Scale (COVID-VCBS) is a unidimensional, reliable and invariant measure of conspiracy beliefs about the COVID-19 vaccines among 13 Latin American countries. The results may be useful to guide future MI studies among different countries.

## Data Availability Statement

The datasets presented in this study can be found in online repositories. The names of the repository/repositories and accession number(s) can be found in the article/Supplementary Material.

## Ethics Statement

The studies involving human participants were reviewed and approved by Ethics Committee of the Universidad Privada del Norte in Peru (registration number: 20213002). The patients/participants provided their written informed consent to participate in this study.

## Author Contributions

TC-R and PV provided initial conception, organization, and main writing of the text. JV-L analyzed the data and prepared all figures and tables. LV, PV, CC-L, MR-B, MW, CR-J, RP-C, MG, MC, PM, DP, RM-H, AS-P, ML-R, AF, DP-C, IC-R, RC, BT, WA, and OP were involved in data collection for their respective countries, acted as consultants, contributors to research design, data analysis, text writing, and read and approved the draft. All authors contributed to the article and approved the submitted version.

## Funding

This research was funded by a COVID-19 crisis seed grant from the Universidad Privada del Norte to TC-R.

## Conflict of Interest

The authors declare that the research was conducted in the absence of any commercial or financial relationships that could be construed as a potential conflict of interest.

## Publisher's Note

All claims expressed in this article are solely those of the authors and do not necessarily represent those of their affiliated organizations, or those of the publisher, the editors and the reviewers. Any product that may be evaluated in this article, or claim that may be made by its manufacturer, is not guaranteed or endorsed by the publisher.

## References

[1] Gates B. Responding to Covid-19—a once-in-a-century pandemic? N Engl J Med. (2020) 382:1677–9. 10.1056/NEJMp2003762 32109012

[2] Coronavirus Resource Center COVID-19 Dashboard by the Center for Systems Science and Engineering (CSSE) at Johns Hopkins University (JHU) (2021). Available online at: https://coronavirus.jhu.edu/map.html (accessed January 10, 2022).

[3] Sallam M Dababseh D Eid H Hasan H Taim D Al-Mahzoum K. Low covid-19 vaccine acceptance is correlated with conspiracy beliefs among university students in Jordan. Int J Environ Res Public Health. (2021) 18:2407. 10.3390/ijerph18052407 33804558 PMC7967761

[4] Plans-Rubió P. The vaccination coverage required to establish herd immunity against influenza viruses. Prev Med. (2012) 55:72–7. 10.1016/j.ypmed.2012.02.015 22414740

[5] Hodgson D Flasche S Jit M Kucharski AJ Centre for Mathematical Modelling of Infectious Disease (CMMID) COVID-19 Working Group. The potential for vaccination-induced herd immunity against the SARS-CoV-2 B 11 7 variant. Euro Surveill. (2021) 26:2100428. 10.2807/1560-7917.ES.2021.26.20.2100428 34018481 PMC8138959

[6] Bolotin S Wilson S Murti M. Achieving and sustaining herd immunity to SARS-CoV-2. CMAJ. (2021) 193:E1089. 10.1503/cmaj.210892 34281966 PMC8315197

[7] Kwok KO Lai F Wei WI Wong SYS Tang JW. Herd immunity–estimating the level required to halt the COVID-19 epidemics in affected countries. J Infect. (2020) 80:e32–3. 10.1016/j.jinf.2020.03.027 32209383 PMC7151357

[8] Bangerter A Krings F Mouton A Gilles I Green EG Clémence A. Longitudinal investigation of public trust in institutions relative to the 2009 H1N1 pandemic in Switzerland. PLoS ONE. (2012) 7:e49806. 10.1371/journal.pone.0049806 23185444 PMC3504102

[9] Thangaraju P Venkatesan S WHO. Ten threats to global health in 2019: antimicrobial resistance. Cukurova Med J. (2019) 44:1150–1. 10.17826/cumj.51181032242168

[10] Sallam M. COVID-19 vaccine hesitancy worldwide: a concise systematic review of vaccine acceptance rates. Vaccines. (2021) 9:160. 10.3390/vaccines9020160 33669441 PMC7920465

[11] Arshad MS Hussain I Mahmood T Hayat K Majeed A Imran I. National survey to assess the COVID-19 vaccine-related conspiracy beliefs, acceptability, preference, and willingness to pay among the general population of Pakistan. Vaccines. (2021) 9:720. 10.3390/vaccines9070720 34358136 PMC8310108

[12] Argote P Barham E Daly SZ Gerez JE Marshall J Pocasangre O. The shot, the message, and the messenger: COVID-19 vaccine acceptance in Latin America. NPJ Vaccines. (2021) 6:1–9. 10.1038/s41541-021-00380-x 34593822 PMC8484594

[13] Urrunaga-Pastor D Herrera-Añazco P Uyen-Cateriano A Toro-Huamanchumo CJ Rodriguez-Morales AJ Hernandez AV. Prevalence and factors associated with parents' non-intention to vaccinate their children and adolescents against COVID-19 in Latin America and the Caribbean. Vaccines. (2021) 9:1303. 10.3390/vaccines9111303 34835233 PMC8624413

[14] Loomba S de Figueiredo A Piatek SJ de Graaf K Larson HJ. Measuring the impact of COVID-19 vaccine misinformation on vaccination intent in the UK and USA. Nat Hum Behav. (2021) 5:337–48. 10.1038/s41562-021-01056-1 33547453

[15] Sallam M Dababseh D Eid H Al-Mahzoum K Al-Haidar A Taim D. High rates of COVID-19 vaccine hesitancy and its association with conspiracy beliefs: a study in Jordan and Kuwait among other Arab countries. Vaccines. (2021) 9:42. 10.3390/vaccines9010042 33445581 PMC7826844

[16] Stein RA Ometa O Shetty SP Katz A Popitiu MI Brotherton R. Conspiracy theories in the era of COVID-19: a tale of two pandemics. Int J Clin Pract. (2021) 75:e13778. 10.1111/ijcp.13778 33480171 PMC7995222

[17] Wirawan GBS Mahardani PNTY Cahyani MRK Laksmi NLPSP Januraga PP. Conspiracy beliefs and trust as determinants of COVID-19 vaccine acceptance in Bali, Indonesia: cross-sectional study. Pers Individ Dif. (2021) 180:110995. 10.1016/j.paid.2021.110995 34007092 PMC8118669

[18] Swami V Voracek M Stieger S Tran US Furnham A. Analytic thinking reduces belief in conspiracy theories. Cognition. (2014) 133:572–85. 10.1016/j.cognition.2014.08.006 25217762

[19] Taylor S Landry CA Paluszek MM Groenewoud R Rachor GS Asmundson GJ. proactive approach for managing COVID-19: the importance of understanding the motivational roots of vaccination hesitancy for SARS-CoV2. Front Psychol. (2020) 11:2890. 10.3389/fpsyg.2020.575950 33192883 PMC7604422

[20] Ball P Maxmen A. The epic battle against coronavirus misinformation and conspiracy theories. Nature. (2020) 581:371–5. 10.1038/d41586-020-01452-z 32461658

[21] Sallam M Dababseh D Yaseen A Al-Haidar A Ababneh NA Bakri FG. Conspiracy beliefs are associated with lower knowledge and higher anxiety levels regarding COVID-19 among students at the University of Jordan. Int J Environ Res Public Health. (2020) 17:4915. 10.3390/ijerph17144915 32650409 PMC7399915

[22] Allington D Duffy B Wessely S Dhavan N Rubin J. Health-protective behaviour, social media usage and conspiracy belief during the COVID-19 public health emergency. Psychol Med. (2021) 51:1763–9. 10.1017/S003329172000224X 32513320 PMC7298098

[23] Earnshaw VA Eaton LA Kalichman SC Brousseau NM Hill EC Fox AB. COVID-19 conspiracy beliefs, health behaviors, and policy support. Transl Behav Med. (2020) 10:850–6. 10.1093/tbm/ibaa090 32910819 PMC7499784

[24] Hornsey MJ Chapman CM Alvarez B Bentley S Salvador Casara BG Crimston CR. To what extent are conspiracy theorists concerned for self versus others? A COVID-19 test case. Eur J Soc Psychol. (2021) 51:285–93. 10.1002/ejsp.2737 33821057 PMC8014880

[25] Bertin P Nera K Delouvée S. Conspiracy beliefs, rejection of vaccination, and support for hydroxychloroquine: A conceptual replication-extension in the COVID-19 pandemic context. Front Psychol. (2020) 11:2471. 10.3389/fpsyg.2020.565128 33071892 PMC7536556

[26] Simione L Vagni M Gnagnarella C Bersani G Pajardi D. Mistrust and beliefs in conspiracy theories differently mediate the effects of psychological factors on propensity for COVID-19 vaccine. Front Psychol. (2021) 12:683684. 10.3389/fpsyg.2021.683684 34305736 PMC8292632

[27] Ceron W Sanseverino GG de-Lima-Santos MF Quiles MG. COVID-19 fake news diffusion across Latin America. Soc Netw Anal Min. (2021) 11:1–20. 10.1007/s13278-021-00753-z 34025818 PMC8132282

[28] Brotherton R French CC Pickering AD. Measuring belief in conspiracy theories: the generic conspiracist beliefs scale. Front Psychol. (2013) 4:279. 10.3389/fpsyg.2013.00279 23734136 PMC3659314

[29] Bruder M Haffke P Neave N Nouripanah N Imhoff R. Measuring individual differences in generic beliefs in conspiracy theories across cultures: conspiracy mentality questionnaire. Front Psychol. (2013) 4:225. 10.3389/fpsyg.2013.00225 23641227 PMC3639408

[30] Lantian A Muller D Nurra C Douglas KM. Measuring belief in conspiracy theories: Validation of a French and English single-item scale. Int Rev Soc Psychol. (2016) 29:1–14. 10.5334/irsp.8

[31] Jolley D Douglas KM. The effects of anti-vaccine conspiracy theories on vaccination intentions. PLoS ONE. (2014) 9:e89177. 10.1371/journal.pone.0089177 24586574 PMC3930676

[32] Shapiro GK Holding A Perez S Amsel R Rosberger Z. Validation of the vaccine conspiracy beliefs scale. Papillomavirus Res. (2016) 2:167–72. 10.1016/j.pvr.2016.09.001 29074176 PMC5886898

[33] Sijtsma K Junker BW. Item response theory: past performance, present developments, and future expectations. Behaviormetrika. (2006) 33:75–102. 10.2333/bhmk.33.75

[34] Hambleton RK Jones RW. Comparison of classical test theory and item response theory and their applications to test development. Educ Meas. (1993) 12:38–47. 10.1111/j.1745-3992.1993.tb00543.x

[35] Volk C Rosenstiel S Demetriou Y Sudeck G Thiel A Wagner W. Health-related fitness knowledge in adolescence: evaluation of a new test considering different psychometric approaches (CTT and IRT). Ger J Exerc Sport Res. (2021) 52:11–23. 10.1007/s12662-021-00735-5

[36] Crocker L Algina J. Introduction to Classical and Modern Test Theory. Fort Worth: Holt, Rinehart and Winston (1986).

[37] Lord FM. Applications of Item Response Theory. Hillsdale, NJ: Erlbaum (1980).

[38] Samejima F. Graded response model. In: van der Linden WJ Hambleton RK editors. Handbook of Modern Item Response Theory. New York, NY: Springer (1996). p. 85–100.

[39] Jiang S Wang C Weiss DJ. Sample size requirements for estimation of item parameters in the multidimensional graded response model. Front Psychol. (2016) 7:109. 10.3389/fpsyg.2016.00109 26903916 PMC4746434

[40] Auné SE Abal FJ Attorresi HF. Application of the graded response model to a scale of empathie behavior. Int J Psychol Res. (2019) 12:49–56. 10.21500/20112084.3753 32612787 PMC7110165

[41] Raykov T Dimitrov DM Marcoulides GA Harrison M. On the connections between item response theory and classical test theory: a note on true score evaluation for polytomous items via item response modeling. Educ Psychol Meas. (2019) 79:1198–209. 10.1177/0013164417745949 31619845 PMC6777063

[42] Cordonier L Cafiero F Bronner G. Why are conspiracy theories more successful in some countries than in others? An exploratory study on Internet users from 22 Western and non-Western countries. Soc Sci Inf. (2021) 60:436–56. 10.1177/05390184211018961

[43] Sternisko A Cichocka A Cislak A Van Bavel JJ. National narcissism predicts the belief in and the dissemination of conspiracy theories during the COVID-19 pandemic: evidence from 56 countries. Pers Soc Psychol Bull. (2021) 1–18. 10.1177/01461672211054947 34872399 PMC9684064

[44] Hornsey MJ Harris EA Fielding KS. The psychological roots of anti-vaccination attitudes: a 24-nation investigation. Health Psychol. (2018) 37:307–15. 10.1037/hea0000586 29389158

[45] Boer D Hanke K He J. On detecting systematic measurement error in cross-cultural research: a review and critical reflection on equivalence and invariance tests. J Cross Cult Psychol. (2018) 49:713–34. 10.1177/0022022117749042

[46] Millsap RE. Statistical Approaches to Measurement Invariance. New York: Routledge (2012).

[47] Chen FF. What happens if we compare chopsticks with forks? The impact of making inappropriate comparisons in cross-cultural research. J Pers Soc Psychol. (2008) 95:1005–18. 10.1037/a0013193 18954190

[48] Vandenberg RJ Lance CE. A review and synthesis of the measurement invariance literature: suggestions, practices, and recommendations for organizational research. Organ Res Methods. (2000) 3:4–70. 10.1177/109442810031002

[49] Scholten S Velten J Bieda A Zhang XC Margraf J. Testing measurement invariance of the depression, anxiety, and stress scales (DASS-21) across four countries. Psychol Assess. (2017) 29:1376–90. 10.1037/pas0000440 28125249

[50] Davidov E Meuleman B Cieciuch J Schmidt P Billiet J. Measurement equivalence in cross-national research. Annu Rev Sociol. (2014) 40:55–75. 10.1146/annurev-soc-071913-043137

[51] Lomazzi V. Using alignment optimization to test the measurement invariance of gender role attitudes in 59 countries. Methods Data Anal. (2018) 12:77–103. 10.12758/mda.2017.09

[52] Steenkamp JBE Baumgartner H. Assessing measurement invariance in cross-national consumer research. J Consum Res. (1998) 25:78–90. 10.1086/209528

[53] Davidov E. Testing for comparability of human values across countries and time with the third round of the European social survey. Int J Comp Sociol. (2010) 51:171–91. 10.1177/0020715210363534

[54] Asparouhov T Muthén B. Multiple-group factor analysis alignment. Struct Equ Modeling. (2014) 21:495–508. 10.1080/10705511.2014.919210

[55] Muthén B Asparouhov T. BSEM measurement invariance analysis. Mplus Web Notes. (2013) 17:1–48. Available online at: https://www.statmodel.com/examples/webnotes/webnote17.pdf

[56] Marsh HW Guo J Parker PD Nagengast B Asparouhov T Muthén B. What to do when scalar invariance fails: the extended alignment method for multi-group factor analysis comparison of latent means across many groups. Psychol Methods. (2018) 23:524–45. 10.1037/met0000113 28080078

[57] Schlipphak B Bollwerk M Back M. Beliefs in conspiracy theories (CT): the role of country context. Political Res Exch. (2019) 3:e1949358. 10.1080/2474736X.2021.1949358

[58] van Prooijen JW Van Vugt M. Conspiracy theories: evolved functions and psychological mechanisms. Perspect Psychol Sci. (2018) 13:770–88. 10.1177/1745691618774270 30231213 PMC6238178

[59] Burki T. COVID-19 in Latin America. Lancet Infect Dis. (2020) 20:547–8. 10.1016/S1473-3099(20)30303-0 32311323 PMC7164892

[60] Pablos-Méndez A Vega J Aranguren FP Tabish H Raviglione MC. Covid-19 in Latin America. BMJ. (2020) 370:m2939. 10.1136/bmj.m2939 32718938

[61] Segovia J Pontarollo N Orellana M. Discontent with democracy in Latin America. Cam J Reg Econ Soc. (2021) 14:417–38. 10.1093/cjres/rsab020 PMC8500101 40504200

[62] Andrade G. COVID-19 vaccine hesitancy, conspiracist beliefs, paranoid ideation and perceived ethnic discrimination in a sample of University students in Venezuela. Vaccine. (2021) 39:6837–42. 10.1016/j.vaccine.2021.10.037 34711439 PMC8531467

[63] Jensen EA Pfleger A Herbig L Wagoner B Lorenz L Watzlawik M. What drives belief in vaccination conspiracy theories in Germany? Front Commun. (2021) 6:105. 10.3389/fcomm.2021.678335

[64] Ruiz JB Bell RA. Predictors of intention to vaccinate against COVID-19: results of a nationwide survey. Vaccine. (2021) 39:1080–6. 10.1016/j.vaccine.2021.01.010 33461833 PMC7794597

[65] Zelič Ž Berič M Grum DK. Examining the role of Covid-19 conspiracy beliefs in predicting vaccination intentions, preventive behavior and willingness to share opinions about the coronavirus. Stud Psychol. (2022) 64:136–53. 10.31577/sp.2022.01.844

[66] Ato M López-García JJ Benavente A. Un sistema de clasificación de los diseños de investigación en psicología. Anal Psicol. (2013) 29:1038–59. 10.6018/analesps.29.3.178511

[67] Caycho-Rodríguez T Valencia PD Vilca LW Lee SA Carbajal-León C Vivanco-Vidal A. COVID-19 bereavement in ten Latin American countries: measurement invariance of the pandemic grief scale and its relation to suicidal ideation. Omega. (2021) 1–29. 10.1177/00302228211048566 34666552 PMC10647883

[68] Pierce BS Perrin PB Tyler CM McKee GB Watson JD. The COVID-19 telepsychology revolution: a national study of pandemic-based changes in US mental health care delivery. Am Psychol. (2021) 76:14–25. 10.1037/amp0000722 32816503

[69] Caycho-Rodríguez T Valencia PD Vilca LW Carbajal-León C Vivanco-Vidal A Saroli-Araníbar D. Prevalence and predictors of intention to be vaccinated against COVID-19 in thirteen Latin American and Caribbean countries. Trends Psychol. (2022) 1–25. 10.1007/s43076-022-00170-x PMC8937005 40479247

[70] Latkin C Dayton LA Yi G Konstantopoulos A Park J Maulsby C. COVID-19 vaccine intentions in the United States, a social-ecological framework. Vaccine. (2021) 39:2288–94. 10.1016/j.vaccine.2021.02.058 33771392 PMC7945864

[71] Our World in Data (2021). Coronavirus (COVID-19) Vaccinations (2022). Available online at: https://ourworldindata.org/covid-vaccinations (accessed January 19, 2022).

[72] Penfield RD Giacobbi JrPR. Applying a score confidence interval to Aiken's item content-relevance index. Meas Phys Educ Exerc Sci. (2004) 8:213–25. 10.1207/s15327841mpee0804_3

[73] Caycho T. Aportes a la cuantificación de la validez de contenido de cuestionarios en enfermería. Rev Cubana Enferm. (2018) 34:262–4. Available online at: http://revenfermeria.sld.cu/index.php/enf/article/view/2779/343

[74] Ventura-León J. De regreso a la validez basada en el contenido. Adicciones. (2019) 1–3. 10.20882/adicciones.1213 31018009

[75] Aiken LR. Content validity and reliability of single items or questionnaires. Educ Psychol Meas. (1980) 40:955–9. 10.1177/001316448004000419

[76] Ferrando PJ Anguiano-Carrasco C. El análisis factorial como técnica de investigación en psicología [Factor analysis as a technique in psychological research]. Papeles del Psicólogo. (2010) 31:18–33. Available online at: https://www.redalyc.org/pdf/778/77812441003.pdf

[77] Yuan K-H Bentler PM. Three likelihood-based methods for mean and covariance structure analysis with nonnormal missing data. Sociol Methodol. (2000) 30:165–200. 10.1111/0081-1750.00078

[78] Hu L Bentler PM. Cutoff criteria for fit indexes in covariance structure analysis: Conventional criteria versus new alternatives. Struct Equ Modeling. (1999) 6:1–55. 10.1080/10705519909540118

[79] Dimitrov DM. Testing for factorial invariance in the context of construct validation. Meas Eval Couns Dev. (2010) 43:121–49. 10.1177/0748175610373459

[80] Fischer R Karl JA. A primer to (cross-cultural) multi-group invariance testing possibilities in R. Front Psychol. (2019) 10:1507. 10.3389/fpsyg.2019.01507 31379641 PMC6657455

[81] Muthén B Asparouhov T. IRT studies of many groups: the alignment method. Front Psychol. (2014) 5:978. 10.3389/fpsyg.2014.00978 25309470 PMC4162377

[82] Furr. Psychometrics: An Introduction (3rd ed.). Thousand Oaks, CA: SAGE (2018).

[83] Edelen MO Reeve BB. Applying item response theory (IRT) modeling to questionnaire development, evaluation, and refinement. Qual Life Res. (2007) 16:5–18. 10.1007/s11136-007-9198-0 17375372

[84] Fraley RC Waller NG Brennan KA. An item response theory analysis of self-report measures of adult attachment. J Pers Soc Psychol. (2000) 78:350–65. 10.1037/0022-3514.78.2.350 10707340

[85] Brown TA. Confirmatory Factor Analysis for Applied Research. 2nd ed. New York, NY: Guilford Press (2015).

[86] Savalei V. A comparison of several approaches for controlling measurement error in small samples. Psychol Methods. (2019) 24:352–70. 10.1037/met0000181 29781637

[87] Hughes S Machan L. It's a conspiracy: covid-19 conspiracies link to psychopathy, Machiavellianism and collective narcissism. Pers Individ Dif. (2021) 171:110559. 10.1016/j.paid.2020.110559 33867616 PMC8035125

[88] Milfont TL Fischer R. Testing measurement invariance across groups: applications in cross-cultural research. Int J Psychol Res. (2010) 3:111–30. 10.21500/20112084.857

[89] Buil I de Chernatony L Martínez E. Methodological issues in cross-cultural research: an overview and recommendations. J Target Meas Anal Mark. (2012) 20:223–34. 10.1057/jt.2012.18

[90] McDonald RP. Test Theory: A Unified Treatment. Mahwah, NJ: L Erlbaum Associates (1999).

[91] Hagell P. Testing rating scale unidimensionality using the principal component analysis (PCA)/t-test protocol with the Rasch model: the primacy of theory over statistics. Open J Stat. (2014) 4:456–65. 10.4236/ojs.2014.46044

[92] Gustafsson J-E Åberg-Bengtsson L. Unidimensionality and interpretability of psychological instruments. In: Embretson SE editor. Measuring Psychological Constructs: Advances in Model-Based Approaches. Washington, DC: American Psychological Association (2010). p. 97–121.

[93] Immekus JC. Multigroup CFA and alignment approaches for testing measurement invariance and factor score estimation: Illustration with the schoolwork-related anxiety survey across countries and gender. Methodol. (2021) 17:22–38. 10.5964/meth.2281

[94] Wong LP Alias H Danaee M Ahmed J Lachyan A Cai CZ. COVID-19 vaccination intention and vaccine characteristics influencing vaccination acceptance: a global survey of 17 countries. Infect Dis Poverty. (2021) 10:1–14. 10.1186/s40249-021-00900-w 34620243 PMC8496428

[95] Munck I Barber C Torney-Purta J. Measurement invariance in comparing attitudes toward immigrants among youth across Europe in 1999 and 2009: the alignment method applied to IEA CIVED and ICCS. Sociol Methods Res. (2018) 47:687–728. 10.1177/0049124117729691

[96] Cieciuch J Davidov E Schmidt P. Alignment optimization: estimation of the most trustworthy means in cross-cultural studies even in the presence of noninvariance. In: Davidov E Schmidt P Billiet J Meuleman B editors. Cross-Cultural Analysis: Methods and Applications. New York, NY: Routledge (2018). p. 571–92.

[97] Altamirano VF Bacon SL Baró S Benítez DA Caravello JC Filippa NL. Representaciones Sociales sobre las Vacunas y la Vacunación frente al COVID 19. Revista Cientí*fica Arbitrada de la Fundación MenteClara*. (2021) 6:1–15. 10.32351/rca.v6.252

[98] Marco JJG Pasquín MJÁ Martín SM. Efectividad y seguridad de las vacunas para el SARS-CoV-2 actualmente disponibles. FMC. (2021) 28:442–51. 10.1016/j.fmc.2021.07.001 34611388 PMC8483629

[99] Wood MJ Douglas KM. What about building 7? a social psychological study of online discussion of 9/11 conspiracy theories. Front Psychol. (2013) 4:409. 10.3389/fpsyg.2013.00409 23847577 PMC3703523

[100] Anderson RM Vegvari C Truscott J Collyer BS. Challenges in creating herd immunity to SARS-CoV-2 infection by mass vaccination. Lancet. (2020) 396:1614–6. 10.1016/S0140-6736(20)32318-7 33159850 PMC7836302

[101] Meda-Lara RM Juárez-Rodríguez P Carrasco-Tapias NE Barrales-Díaz CR Palomera-Chávez A González-Díaz E. Precautionary behaviors during the second and third phases of the COVID-19 pandemic: comparative study in the Latin American population. Int J Environ Res Public Health. (2021) 18:6882. 10.3390/ijerph18136882 34206907 PMC8297200

[102] Cerda AA García LY. Willingness to Pay for a COVID-19 Vaccine. Appl Health Econ Health Policy. (2021) 19:343–51. 10.1007/s40258-021-00644-6 33619688 PMC7899739

[103] Zhang Y Luo X Ma ZF. Willingness of the general population to accept and pay for COVID-19 vaccination during the early stages of COVID-19 pandemic: a nationally representative survey in mainland China. Hum Vaccin Immunother. (2021) 17:1622–7. 10.1080/21645515.2020.1847585 33606600 PMC8115554

[104] Rosiello DF Ferreto LE Aburto JT Rojas JE Enitan SS Yomi AR. Acceptance of COVID-19 vaccination at different hypothetical efficacy and safety levels in ten countries in Asia, Africa, and South America. Narra J. (2021) 1:e55. 10.52225/narra.v1i3.55 PMC10914086 38450212

[105] Vega-Dienstmaier JM. Teorías de conspiración y desinformación entorno a la epidemia de la COVID-19. Rev Neuropsiquiatr. (2020) 83:135–7. 10.20453/rnp.v83i3.3792

[106] Mostajo-Radji MA. Pseudoscience in the times of crisis: how and why chlorine dioxide consumption became popular in Latin America during the COVID-19 pandemic. Front Polit Sci. (2021) 3:25. 10.3389/fpos.2021.621370

[107] Biddlestone M Green R Douglas KM. Cultural orientation, power, belief in conspiracy theories, and intentions to reduce the spread of COVID-19. Br J Soc Psychol. (2020) 59:663–73. 10.1111/bjso.12397 32592420 PMC7361833

[108] Plohl N Musil B. Modeling compliance with COVID-19 prevention guidelines: The critical role of trust in science. Psychol Health Med. (2021) 26:1–12. 10.1080/13548506.2020.1772988 32479113

[109] Tonković M Dumančić F Jelić M Biruški DC. Who believes in COVID-19 conspiracy theories in Croatia? prevalence and predictors of conspiracy beliefs. Front Psychol. (2021) 12:643568. 10.3389/fpsyg.2021.643568 34220613 PMC8249866

[110] Scrima F Miceli S Caci B Cardaci M. The relationship between fear of COVID-19 and intention to get vaccinated. The serial mediation roles of existential anxiety and conspiracy beliefs. Pers Individ Dif. (2022) 184:111188. 10.1016/j.paid.2021.111188 34393312 PMC8354796

[111] Salali GD Uysal MS. COVID-19 vaccine hesitancy is associated with beliefs on the origin of the novel coronavirus in the UK and Turkey. Psychol Med. (2020) 1–3. 10.1017/S0033291720004067 33070804 PMC7609204

[112] Bierwiaczonek K Gundersen AB Kunst JR. The role of conspiracy beliefs for COVID-19 health responses: a meta-analysis. Curr Opin Psychol. (2022) 46:101346. 10.1016/j.copsyc.2022.101346 35486966 PMC8978448

[113] Lewandowsky S Gignac GE Oberauer K. The role of conspiracist ideation and worldviews in predicting rejection of science. PLoS ONE. (2013) 8:e75637. 10.1371/journal.pone.0075637 24098391 PMC3788812

[114] Eberhardt J Ling J. Predicting COVID-19 vaccination intention using protection motivation theory and conspiracy beliefs. Vaccine. (2021) 39:6269–75. 10.1016/j.vaccine.2021.09.010 34535313 PMC8421109

[115] Yang Z Luo X Jia H. Is it all a conspiracy? Conspiracy theories and people's attitude to COVID-19 vaccination. Vaccines. (2021) 9:1051. 10.3390/vaccines9101051 34696159 PMC8540771

[116] Daly M Robinson E. Longitudinal changes in psychological distress in the UK from 2019 to September 2020 during the COVID-19 pandemic: Evidence from a large nationally representative study. Psychiatry Res. (2021) 300:113920. 10.1016/j.psychres.2021.113920 33882397 PMC9755113

[117] González-Melado FJ Di Pietro ML. La vacuna frente a la COVID-19 y la confianza institucional. Enferm Infecc Microbiol Clin. (2021) 39:510–5. 10.1016/j.eimc.2020.08.001 33069493 PMC7834478

[118] Jennings W Stoker G Bunting H Valgarð*sson VO Gaskell J Devine D. Lack of trust, conspiracy beliefs, and social media use predict COVID-19 vaccine hesitancy. Vaccines. (2021) 9:593. 10.3390/vaccines9060593 34204971 PMC8226842

[119] Seddig D Maskileyson D Davidov E Ajzen I Schmidt P. Correlates of COVID-19 vaccination intentions: attitudes, institutional trust, fear, conspiracy beliefs, and vaccine skepticism. Soc Sci Med. (2022) 302:114981. 10.1016/j.socscimed.2022.114981 35512613 PMC9017059

[120] Vezzoni C Sani GMD Chiesi AM Ladini R Biolcati F Guglielmi S. Where does the coronavirus come from? On the mechanisms underlying the endorsement of conspiracy theories on the origin of SARS-CoV-2. Ital Polit Sci Rev. (2022) 52:51–65. 10.1017/ipo.2021.19

[121] Pivetti M Melotti G Bonomo M Hakoköngäs E. Conspiracy beliefs and acceptance of COVID-vaccine: an exploratory study in Italy. Soc Sci. (2021) 10:108. 10.3390/socsci10030108

[122] Stecula DA Pickup M. How populism and conservative media fuel conspiracy beliefs about COVID-19 and what it means for COVID-19 behaviors. Res Polit. (2021) 8:2053168021993979. 10.1177/2053168021993979

[123] McCarthy M Murphy K Sargeant E Williamson H. Examining the relationship between conspiracy theories and COVID-19 vaccine hesitancy: a mediating role for perceived health threats, trust, and anomie? Anal Soc Issues Public Policy. (2022) 22:106–29. 10.1111/asap.12291

[124] Caycho-Rodríguez T Ventura-León J Valencia PD Vilca LW Carbajal-León C Reyes-Bossio M. What is the support for conspiracy beliefs about COVID-19 vaccines in Latin America? a prospective exploratory study in 13 countries. Front Psychol. (2022) 13:855713. 10.3389/fpsyg.2022.855713 35602688 PMC9120924

